# Metataxonomic Analysis of Grape Microbiota During Wine Fermentation Reveals the Distinction of Cyprus Regional *terroirs*

**DOI:** 10.3389/fmicb.2021.726483

**Published:** 2021-09-22

**Authors:** Eleni Kamilari, Minas Mina, Christos Karallis, Dimitrios Tsaltas

**Affiliations:** ^1^Department of Agricultural Sciences, Biotechnology, and Food Science, Cyprus University of Technology, Lemesos, Cyprus; ^2^Kyperounda Winery, P. Photiades Group, Nicosia, Cyprus

**Keywords:** wine, fermentation, microbiome, HTS, terroir, amplicon sequencing, 16S rRNA, ITS

## Abstract

Wine production in Cyprus has strong cultural ties with the island’s tradition, influencing local and foreign consumers’ preferences and contributing significantly to Cyprus’ economy. A key contributor to wine quality and sensorial characteristics development is the microbiota that colonizes grapes and performs alcoholic fermentation. Still, the microbial patterns of wines produced in different geographic regions (*terroir*) in Cyprus remain unknown. The present study investigated the microbial diversity of five *terroirs* in Cyprus, two from the PGI Lemesos region [Kyperounta (PDO Pitsilia) and Koilani (PDO Krasochoria)], and three from the PGI Pafos region [Kathikas (PDO Laona Akamas), Panayia, and Statos (PDO Panayia)], of two grape varieties, Xynisteri and Maratheftiko, using high-throughput amplicon sequencing. Through a longitudinal analysis, we examined the evolution of the bacterial and fungal diversity during spontaneous alcoholic fermentation. Both varieties were characterized by a progressive reduction in their fungal alpha diversity (Shannon index) throughout the process of fermentation. Additionally, the study revealed a distinct separation among different *terroirs* in total fungal community composition (beta-diversity) for the variety Xynisteri. Also, Kyperounta *terroir* had a distinct total fungal beta-diversity from the other *terroirs* for Maratheftiko. Similarly, a significant distinction was demonstrated in total bacterial diversity between the PGI Lemesos region and the PGI Pafos *terroirs* for grape juice of the variety Xynisteri. Pre-fermentation, the fungal diversity for Xynisteri and Maratheftiko was dominated by the genera *Hanseniaspora*, *Aureobasidium*, *Erysiphe*, *Aspergillus*, *Stemphylium*, *Penicillium*, *Alternaria*, *Cladosporium*, and *Mycosphaerella*. During and post-fermentation, the species *Hanseniaspora nectarophila*, *Saccharomyces cerevisiae*, *Hanseniaspora guilliermondii*, and *Aureobasidium pullulans*, became the predominant in most must samples. Regarding the bacterial diversity, *Lactobacillus* and *Streptococcus* were the predominant genera for both grape varieties in all stages of fermentation. During fermentation, an increase was observed in the relative abundance of some bacteria, such as *Acetobacter*, *Gluconobacter*, and *Oenococcus oeni*. Finally, the study revealed microbial biomarkers with statistically significant higher relative representation, associated with each geographic region and each grape variety, during the different stages of fermentation. The present study’s findings provide an additional linkage between the grape microbial community and the wine *terroir*.

## Introduction

Cyprus has been a wine-producing country for centuries, and nowadays, the local wine industry is ranked among the 50 greatest wine producers worldwide (Wine Production (ton), 2015) with an annual production of 10 ml, more than 100 small and four big wineries exist in the island, located in hillside villages. Additionally, there are about 15 indigenous grape varieties, from which Xynisteri, Mavro, Maratheftiko, and Ofthalmo are the most extensively cultivated. The majority of the Cyprus wines are produced by native varieties, contributing to their unique and distinct aroma and flavors. The “*terroir* approach” is now more evident, with “Single Vineyard” wines released by the producers.

**FIGURE 1:**
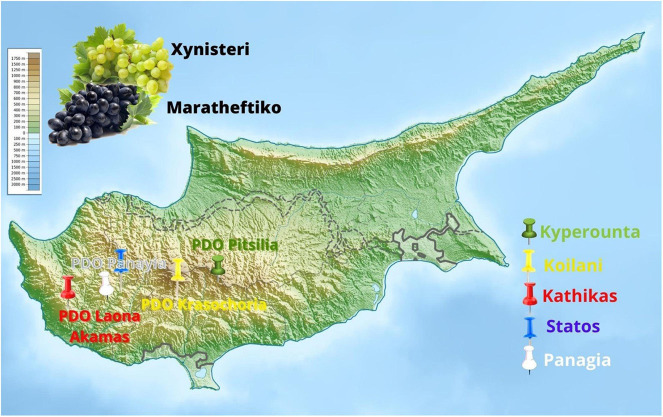
Map of wine grape sampling sites.

The exclusive sensorial characteristics of wines are influenced by the contribution of regional environmental factors, including topography, climatic conditions, soil, different varieties, and microbial patterns, generally known as “*terroir*” (Bokulich et al., 2013; Anagnostopoulos et al., 2019). Differences in topoclimate, soil, and vines distinguish a plethora of *terroirs* in Cyprus, providing a specific character in different *terroir’* produced wines (Kokkinofta et al., 2017). The characterization of Cyprus wines’ typicity is essential for defining protected designation of origin (PDO) and protected geographical indication (PGI) wines’ designation. The European Union (EU) has established the PDO and PGI labels to safeguard local products authenticity. In Cyprus, there are five PDO recognized regions: (i) Commandaria, (ii) Krasochoria Lemesou, and (iii) Pitsilia, located in the Lemesos PGI region, (iv) Laona Akamas, and (v) Vouni Panayia-Ampelitis, located in the Paphos PGI region (Figure 1). These labels are critical contributors to consumers’ preferences and the economic appreciation of local wines. The winemakers and the market need to identify and maintain the microbial community that influence *terroir* and affect wines’ sensorial characteristics and quality. This is especially important for spontaneously fermented wines, in which the native microbiota contributes to the wines’ unique flavors and aromas (Ballantyne et al., 2019).

A key contributor to wine *terroir* is the grape microbiota. Specific members of the regional microbial communities guide alcoholic fermentation (AF) producing metabolites that affect the wines’ sensorial characteristics and quality. The process of fermentation is dynamic, leading to modifications in microbial diversity (Wang et al., 2021). Traditionally, the spontaneous fermentation process has been applied in winemaking, according to which no extra microbes or chemical compounds are added pre-fermentation. Under these conditions, the wine microbiota is considered unpredictable. Nowadays, apart from the native microbiota, selected species are added as starter cultures to control the processes of AF, such as *Saccharomyces cerevisiae*, and of malolactic fermentation (MLF), such as *Oenococcus oeni* and *Lactobacillus plantarum* (De Benedictis et al., 2011; Berbegal et al., 2016, 2017; Tristezza et al., 2016; Petruzzi et al., 2017). More recently, mixtures of yeasts (*S. cerevisiae* and non-Saccharomyces) are commercially available, introducing a controlled additional aromatic diversity to the final products (Jolly et al., 2014; Lleixà et al., 2016). The application of the whole genome sequencing methodology assisted in the selection of possible starters, allowing the identification of the total genome sequences of several *S. cerevisiae* strains (Goffeau et al., 1996; Birkeland et al., 2010; Otero et al., 2010; Akao et al., 2011; Borneman et al., 2016), other *Saccharomyces* species (Naseeb et al., 2018), non-*Saccharomyces* yeasts, such as *Hanseniaspora guilliermondii* (Seixas et al., 2019); *Hanseniaspora vineae* (Giorello et al., 2014)*; Torulaspora delbrueckii* (Tondini et al., 2018), and malolactic bacteria, such as *O. oeni* (Mills et al., 2005). As a result, genes associated with metabolic traits that may influence grapes and wine quality have been revealed.

Over the last decade, several High Throughput sequencing (HTS) studies have been conducted to identify the microbial communities developed in wine and to reveal the contribution of the microbial communities in wines taste and aroma (Bokulich et al., 2012, 2016; Knight et al., 2015; Piao et al., 2015; Pinto et al., 2015; Belda et al., 2016). The studies indicated that pre-fermentation factors associated with the regional *terroir* shape the microbial diversity of wine grapes. During AF, regional fungi, including *S. cerevisiae*, metabolize sugars producing alcohol and secondary metabolites that contribute to wine’s sensorial characteristics (Swiegers et al., 2005; Lambrechts and Pretorius, 2019). In addition to AF, native bacteria, such as *Oenococcus*, *Lactobacillus*, *Pediococcus*, and *Leuconostoc*, convert malic acid to lactic acid, influencing wines’ aromas and tastes (Renouf et al., 2005). Post-fermentations, the microbial diversity is formed by species detected in low relative abundances pre-fermentation but can survive this stressful microenvironment. The regional distinction based on the evolution of the microbiological patterns, as revealed through these HTS, increased our understanding regarding the spatiotemporal contribution of the grapes’ microbiome to wines’ typicity.

The determination of the native grape microbial diversity throughout AF unfolds a new horizon for identifying Cyprus wines’ authenticity. Kokkinofta et al. (2017) proved significant discrimination of different grape varieties and produced in different PDOs Cyprus wines based on regional isotopes and elements, using inductively coupled plasma atomic emission spectroscopy (ICP-AES). Our study is the first to discriminate grape wines produced: (i) in diverse PDO regions; and (ii) from the varieties Xynisteri and Maratheftiko, throughout the fermentation process, based on the distinct microbiological patterns.

## Materials and Methods

### Wine Grapes Collection

Wine grapes from the varieties Xynisteri (white) and Maratheftiko (black) were harvested from August until October 2019 from two *terroirs* in Kyperounta village (PDO Pitsilia) and Koilani village (PDO Krasochoria), both located in Lemesos PGI region, and three *terroirs* in Kathikas (PDO Laona Akamas), Panayia and Statos (PDO Panayia), located in Paphos PGI region (Figure 1). Among the five *terroirs*, Kyperounta is at the highest altitude, in 1,134 m elevation above the sea level, followed by Statos in 913 m, Panayia in 900 m, Koilani in 820 m, and finally Kathikas in 655 m. Noteworthy, Panayia and Statos are neighboring villages. From each terroir, four samples were collected, two from one vineyard and another two from a neighbor vineyard. The weight of the samples was about 5 kg grapes/vineyard, and about 8–12 bunches were used. Grapes were fully ripe at 22–23 Brix. Information about samples’ sugar concentration, yeast assimilable nitrogen, pH and total acidity are shown in Supplementary Table 1. Both vineyards were in a radius of less than 1 km apart, showing similar soil and environmental conditions. All vineyards are bush vines, and goblet trained more than 40 years old, with no specific row orientation. Also, the topoclimate between the area samples is the same. The two neighboring vineyards had the same training, microclimate, soil, and crop management (conventional). Some potential differences in crop management, such as spraying, may be attributed to different owners’ practices. Generally, treatments follow very similar patterns as growers follow common local expert advice. Samples were placed in sterile plastic bags and transported to Kyperounta Winery for processing.

### Microvinifications

Whole bunches of grapes were aseptically crushed without any destemming and left for 24 h at 18°C. A 50 ml “pre-fermentation” sample was taken and stored at −80°C until processing. Must was separated from the skins and stems on the second day of fermentation and kept at 18°C for the whole course of the fermentation, for both the varieties. The process of spontaneous fermentation was conducted without sulfites addition. The fermentation course was monitored by daily density measurement with a DMA 35 portable density meter. “During fermentation” sampling was performed when the alcohol production was 4% ABV. The sampling at this stage indicates the exponential phase of fermentation when the yeast population reached its maximum growth (Olivares-Marin et al., 2018). “Post fermentation” sample was taken after the alcohol concentration was higher than 10%, and when for three consecutive days the density readings were stable.

All samples were collected in a sterile 50 ml container and immediately were frozen with dry ice and stored at −80°C until processing.

### Metataxonomic DNA Extraction

Juice and must samples were centrifuged at 14,000 *g* for 30 min at 4°C and washed twice in 2 ml and 300 μl TE buffer, respectively. The DNA pellet was suspended in 450 μl DNeasy^®^ PowerFood^®^ Solution MBL supplemented with 40 mg/ml lysozyme and incubated at 37°C for 30 min, followed by incubation at 65°C for 10 min and 95°C for 10 min. Then the DNA isolation was performed according to DNeasy^®^ PowerFood^®^ Microbial Kit (MoBio Laboratories Inc., Carlsbad, CA, United States) manufacturer’s instructions. The extracted DNA was stored at −20°C until processing. If the concentration of the extracted DNA was lower than 5 ng/μl, or the 260/230 ratio was lower than 1.9, indicating the presence of contaminants, then the process of DNA extraction was repeated with the addition of a small concentration of polivinilpirrolidone and 20 μl of β−mercaptoethanol followed by 1 h incubation at 60°C, before the addition of the lysis buffer. The addition of polivinilpirrolidone removes tannins and polyphenols, whereas β−mercaptoethanol of proteins from the extract.

### Quantification of Total DNA

The total DNA isolated from the juice and must samples was quantified fluorometrically with Qubit 4.0 fluorometer (Invitrogen, Carlsbad, CA) using Qubit dsDNA HS Assay Kit (Invitrogen). The purity of the DNA was evaluated by measuring the ratio of absorbance A260/280 nm and A260/230 nm using a spectrophotometer (NanoDrop Thermo Scientific, United States).

### Barcoded Illumina MiSeq Amplicon Sequencing of Bacterial 16S rRNA Gene and of Fungal ITS Region

The 16S rRNA bacterial gene amplification, the Illumina paired-completion library preparation and sequencing was performed as described previously (Kamilari et al., 2020b). The 16S rRNA bacterial gene was amplified using the primers V3: 50-TCGTCGGCAGCGTCAGATGTGTATAAGAGACAG-30 and V4: 50-GTCTCGTGGGCTCGGAGATGTGTATAAGAGACAG-30, whereas the fungal internal transcribed spacer 1 (ITS1) loci using the primers BITS (5′-NNNNNNNNCTACCTGC GGARGGATCA-3′) and B58S3 (5′-GAGATCCRTTGYTRAAA GTT-3′) with the addition of the overhang adapter sequence, as described by Bokulich et al. (2016). For fungal ITS1 loci amplification and sequencing the “Fungal Metagenomic Sequencing Demonstrated Protocol” provided by Illumina was applied.^1^ The PCR reaction mixture for each reaction was: 2.5 μL template DNA (5 ng/μL); 5 μL of each forward and reverse primers (1 μM) and 12.5 μL 2 × KAPA HiFi HotStart Ready Mix (KAPA Biosystems, United States). PCR amplification was performed in PCR Thermocycler (Bio-Rad, United States) using the following procedure: (a) Denaturation: 95°C for 3 min; (b) Denaturation: 95°C for 30 s; (c) Annealing: 55°C for 30 s; (d) Elongation: 72°C for 30 s; (e) repeat of steps b–d for 25 cycles; (f) Extension: 72°C for 5 min; (g) Hold at 4°C. PCR amplicons purification, evaluation of DNA quantity and quality and amplicons normalization was conducted as describe previously (Papademas et al., 2020). For the sequencing run, both bacterial 16S rRNA gene and fungal ITS1 loci were loaded on MiSeq 600 cycle Reagent Kit v3 (Illumina, United States) (5% PhiX) and run on a MiSeq Illumina sequencing platform.

### Microbiome and Statistical Analysis

Raw fastq sequences were quality filtered, and the diversity indexes Shannon, Simpson, and Chao1 regarding alpha diversity and Bray Curtis dissimilarity regarding beta diversity, were performed using Qiime 2 version 2020.2 (Bolyen et al., 2019), as previously described (Kamilari et al., 2020a). Before diversity analyses, samples were rarefied (Supplementary Table 9). Comparisons of alpha and beta diversity indexes were evaluated separately for the two wine grapes varieties, Xynisteri and Maratheftiko, and for the three stages of the fermentation, pre-, during-, and post-fermentation. To visualize the separation of the bacterial and fungal communities into clusters based on Bray–Curtis dissimilarity distances, the Principal Coordinate Analysis (PCoA) was performed using q2−diversity after samples were rarefied as previously described (Kamilari et al., 2021). To estimate whether sample categories (i) Xynisteri and Maratheftiko; (ii) pre-, during-, and post-fermentation) had statistical differences in their beta microbial diversity, non-parametric permutational analysis of variance (PERMANOVA) (Anderson, 2008) with 999 permutations was applied. To evaluate if the sample categories had differences in alpha and beta diversity, rejecting the null hypothesis, Kruskal-Wallis tests were performed. Additionally, to detect differences in alpha diversity among stages of fermentation, analysis of variance (one-way ANOVA) was applied using the SPSS 20 software (StatSoft Inc., Tulsa, OK, United States). The test used was Least Significant Difference (LSD) at the significance level of 0.05. To assign the taxonomy to the 16S rDNA sequences into OTU the q2−feature−classifier (Bokulich et al., 2018) against the Greengenes 13_8 99% OTUs reference sequences (McDonald et al., 2012) was used, whereas, for fungal ITS loci, the UNITE fungal internal transcribed spacer (ITS) database (8.2 release) (Abarenkov et al., 2020) was applied. The sequences were filtered to remove incomplete taxonomies that failed to be identified to the genus level. For biomarkers discovery, the LEfSe algorithm was used (Segata et al., 2011), with Linear discriminant analysis (LDA) scores greater than 2.0. Taxa with relative abundance < 1% per sample were considered not important to be mentioned.

All raw sequence data in read-pairs format were deposited to the National Centre for Biotechnology Information (NCBI) in Sequence Read Archive (SRA) under BioProject PRJNA731461.

## Results

The present study investigated the contribution of the microbial diversity composition in the distinction of Cyprus wines regional *terroir*, as well as of the wine grape varieties Xynisteri and Maratheftiko, by applying the HTS approach.

### Abundance and Alpha Diversity of the Must Microbiota

Wine grape samples collected from Kyperounta village (PDO Pitsilia) and Koilani village (PDO Krasochoria) (PGI Lemessos region), as well as Kathikas, Panayia, and Statos villages (PDO Panayia, PGI Pafos region) were analyzed separately regarding: (i) the varieties: (a) X ynisteri and (b) Maratheftiko; (ii) The stage of must fermentations: (a) pre-; (b) during, and (c) post-fermentation; and (iii) the microbial communities: (a) bacterial and (b) fungal. In total, from 240 samples, nineteen were excluded from the analysis. Specifically, two pre-fermentation samples from Panayia *terroir* for the variety Xynisteri had a low amount of 16S rDNA during the Library Quantification and Normalization process. The remaining samples were excluded because of a low number of reads passing filter after 16S rDNA library sequencing (eleven samples) and after ITS library sequencing (eight samples).

To evaluate the bacterial diversity of Xynisteri, pre-, during, and post-fermentation, fifty-five examined samples were used as input to the Illumina MiSeq to produce: 2,787,651 high-quality sequencing reads, with an average of 50,684.6 sequencing reads per sample (range = 12,837–405,275, STD = 53,186.7; Supplementary Table 2). High quality sequences were grouped into average number 110.43 OTUs (range = 60–234, SD = 32.8). About the fungal diversity of Xynisteri from fifty-six examined samples 1,445,265 high quality sequencing reads, were produced with an average of 25,808.3 sequencing reads per sample (range = 6,131–430,689, STD = 56,022.2; Supplementary Table 3). High quality sequences were grouped into average number 60.55 OTUs (range = 31–175, SD = 27.7). Results based on the alpha diversity indexes (Shannon, Simpson, and Chao1 estimators) are shown in Supplementary Table 2 for bacterial and Supplementary Table 3 for fungal diversity.

For estimating the bacterial diversity of Maratheftiko, pre-, during, and post-fermentation fifty-one examined samples were sequenced to generate 2,450,489 high-quality sequencing reads, with an average of 48,048.80 sequencing reads per sample (range = 13,475–429,110, STD = 57,464.9; Supplementary Table 4). High-quality sequences were grouped into average number 102.21 OTUs (range = 59–206, SD = 23.8). Regarding the fungal diversity of Maratheftiko from fifty-eight examined samples 1,908,997 high quality sequencing reads, were generated with an average of 32,913.7 sequencing reads per sample (range = 8,586–104,602, STD = 18,964.7; Supplementary Table 5). High quality sequences were grouped into average number 60.93 OTUs (range = 26–146, SD = 26). Results concerning the alpha diversity indexes (Shannon, Simpson, and Chao1 estimators) are shown in Supplementary Table 4 for bacterial and Supplementary Table 5 for fungal diversity.

**FIGURE 2:**
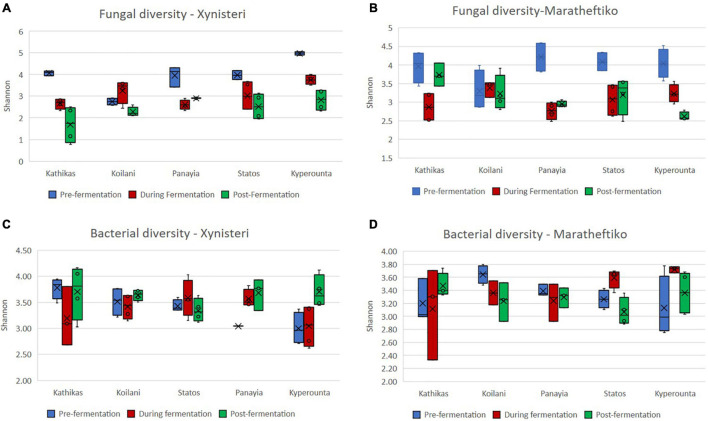
Investigation of the microbial alpha diversity based on the Shannon index in grape juice and must samples for the *terroirs* Kathikas, Koilani, Kyperounta, Panayia, and Statos during the three stages of fermentation (pre-fermentation, during fermentation, and post-fermentation). **(A)** Fungal diversity of the variety Xynisteri; **(B)** fungal diversity of the variety Maratheftiko; **(C)** bacterial diversity for the variety Xynisteri; **(D)** bacterial diversity for the variety Maratheftiko. The blue box-plots represent the fungal or bacterial diversity pre-fermentation. The red box-plots represent the fungal or bacterial diversity during fermentation. The green box-plots represent the fungal or bacterial diversity post-fermentation. The o symbols represent the outliers from the median and x symbols the brand means. Statistical analysis was performed using Kruskal-Wallis test.

**FIGURE 3:**
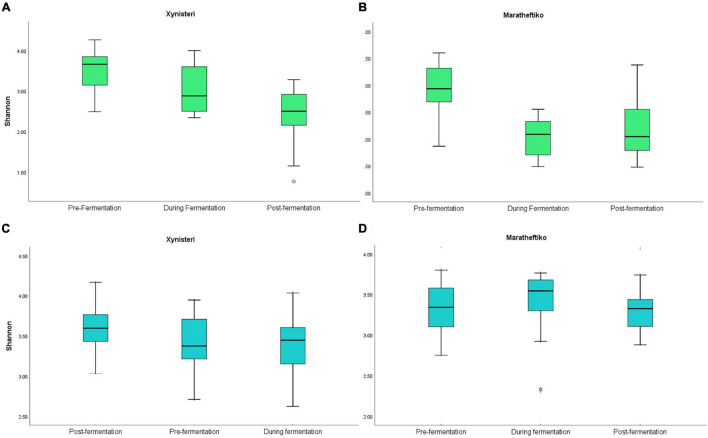
Comparison of the microbial alpha diversity based on the Shannon index during the three stages of fermentation (pre-fermentation, during fermentation, and post-fermentation). **(A)** Fungal diversity of the variety Xynisteri; **(B)** fungal diversity of the variety Maratheftiko; **(C)** bacterial diversity for the variety Xynisteri; **(D)** bacterial diversity for the variety Maratheftiko. The o symbol represents the outliers from the median.

Initially, we compared the fungal alpha diversity (Shannon index) of wine grapes growing in the five *terroirs* of Cyprus, during the three different stages of the fermentation, for the two wine grape varieties, Xynisteri and Maratheftiko. According to Figures 2A,B, pre-fermentation Koilani demonstrated the lowest alpha diversity for both wine grapes varieties. The difference was significant for the variety Xynisteri, based on the Kruskal-Wallis test (*p* = 0.02; Supplementary Table 6). During and post-fermentation, a significant reduction in the alpha diversity was observed in almost all areas for both Xynisteri and Maratheftiko varieties (*p* < 0.05; Figures 3A,B and Supplementary Table 6). Post-fermentation, for the variety Xinisteri, the alpha diversity of Panayia was significantly higher than Kathikas and Koilani, whereas, for the variety Maratheftiko, of Kathikas, Koilani and Panayia compared to Kyperounta, based on the Kruskal-Wallis test (*p* = 0.02; Supplementary Table 6).

Additionally, we compared the bacterial alpha diversity (Shannon index) of the five *terroirs*, pre-, during, and post-fermentation, for Xynisteri and Maratheftiko (Figures 2C,D). Pre-fermentation, for the variety Xynisteri, significantly higher bacterial alpha diversity was indicated for the areas with the lowest altitude, including Kathikas and Koilani, compared to Kyperounta, the village with the highest altitude, based on the Shannon index, by Kruskal-Wallis test (*p* = 0.02). Similarly, Koilani showed the most elevated and Kyperounta the lowest bacterial diversity for the wine grape variety Maratheftiko. During and post-fermentation, no significant differences were detected among the five *terroirs* for both grape varieties and compared to pre-fermentation, based on the Kruskal-Wallis test (Figures 3C,D).

### Microbial Diversity Differentiates Among the Distinct Terroir

To evaluate the existence of different microbial patterns among wine grapes growing in geographically distant areas of Cyprus with different altitude and weather conditions, we tested the microbial beta diversity of the two wine grapes varieties, Xynisteri and Maratheftiko pre-fermentation. In addition, we investigated the presence of distinct bacterial and fungal patterns during and post-fermentation to identify whether the different areas could be discriminated based on their microbiological composition after the completion of the process. To detect differences, we compared the microbiota structure applying the Bray-Curtis dissimilarity.

**FIGURE 4:**
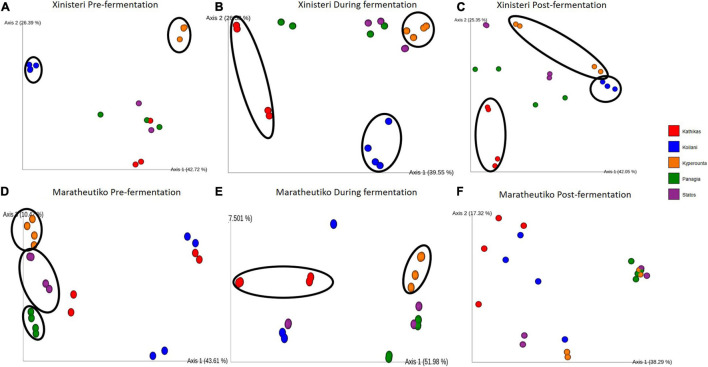
Principal coordinate analysis (PCoA) plot, showing the similarities in the fungal beta diversity according to the Bray-Curtis distance among the individual *terroir*: **(A)** for the variety Xynisteri pre-fermentation; **(B)** for the variety Xynisteri during fermentation; **(C)** for the variety Xynisteri post-fermentation; **(D)** for the variety Maratheftiko pre-fermentation; **(E)** for the variety Maratheftiko during fermentation; **(F)** for the variety Maratheftiko post-fermentation. Red circles represent Kathikas, blue circles represent Koilani, yellow circles represent Kyperounta, green circles represent Panayia and purple circles represent Statos. Ellipses show the separated *terroir*.

**FIGURE 5:**
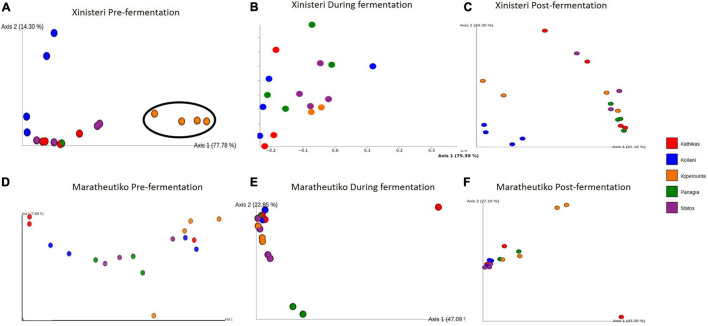
Principal coordinate analysis plot, showing the similarities in the bacterial beta diversity according to the Bray-Curtis distance among the individual *terroir*: **(A)** for the variety Xynisteri pre-fermentation; **(B)** for the variety Xynisteri during fermentation; **(C)** for the variety Xynisteri post-fermentation; **(D)** for the variety Maratheftiko pre-fermentation; **(E)** for the variety Maratheftiko during fermentation; **(F)** for the variety Maratheftiko post-fermentation. Red circles represent Kathikas, blue circles represent Koilani, yellow circles represent Kyperounta, green circles represent Panayia and purple circles represent Statos. Ellipses show the separated *terroir*.

Principal Coordinate analysis plot based on Bray-Curtis distance was developed to depict the separation of the individual *terroir* based on the similarities in the microbial communities. Comparison of the fungal beta diversity for the variety of Xynisteri revealed that pre-fermentation, all the PDO regions were distinct (Figure 4A). PERMANOVA test indicated that the differences were significant (*p* < 0.05; Supplementary Table 7). Noteworthy, Statos and Panayia that belong to the PDO Panayia didn’t show significant differences. The distinction among the different *terroirs* remained significant during the rest of the stages of fermentation for almost all *terroirs* (Figures 4B,C and Supplementary Table 7). Regarding the variety Maratheftiko, the fungal beta-diversity separated Kyperounta, the area found in the higher altitude, from the other *terroir*, pre-, during, and post-fermentation (Figures 4D–F and Supplementary Table 6). PERMANOVA test confirmed a significant difference between Kyperounta and the other *terroir* pre-, during, and post-fermentation (*p* < 0.05). However, the bacterial diversity sufficed to differentiate significantly only the *terroir* Kyperounta, from the rest *terroirs*, for the stage pre-fermentation, variety Xynisteri (Figures 5A–F and Supplementary Table S7). No other significant differences were detected among the individual *terroirs* pre-, during, and post-fermentation for the varieties Xynisteri and Maratheftiko. Furthermore, comparing the Xynisteri and Maratheftiko microbial beta diversity revealed that both bacterial and fungal composition differed significantly during pre- and post-fermentation stages (Supplementary Table 8).

### Taxonomic Composition of Microbial Communities in Grape Juice and Must Samples

**FIGURE 6:**
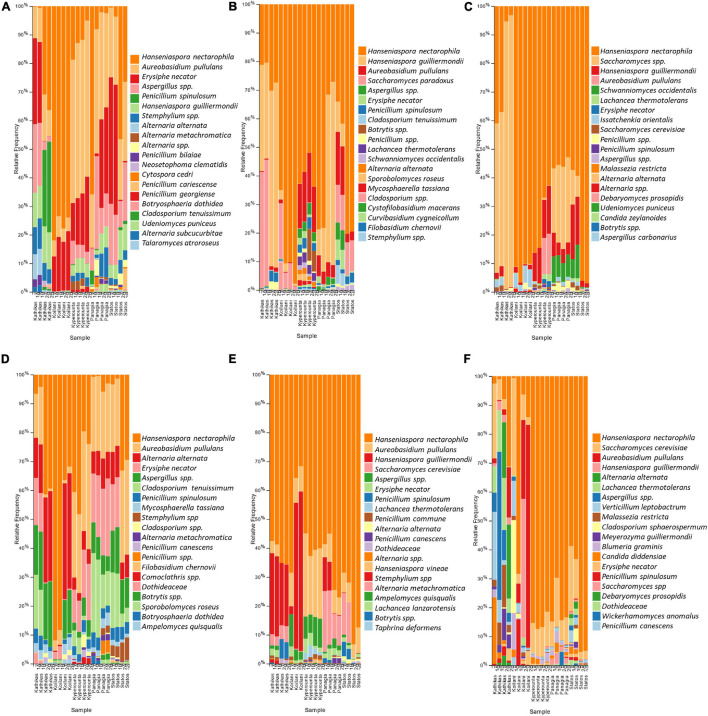
The relative abundance of the twenty most abundant fungi identified at the species level based on ITS loci sequencing for the *terroir* Kathikas, Koilani, Kyperounta, Panayia, and Statos: **(A)** for the variety Xynisteri pre-fermentation; **(B)** for the variety Xynisteri during fermentation; **(C)** for the variety Xynisteri post-fermentation; **(D)** for the variety Maratheftiko pre-fermentation; **(E)** for the variety Maratheftiko during fermentation; **(F)** for the variety Maratheftiko post-fermentation.

The fungal communities of the Cyprus *terroir* for both varieties, Xynisteri and Maratheftiko, from grape juice until the completion of fermentation, according to ITS1 loci sequencing, were characterized mainly by members of the phylum Ascomycota and in lower relative abundances of the phylum Basidiomycota. The most commonly detected species in grape juice belonged to the genera *Hanseniaspora*, including *Hanseniaspora nectarophila* and *H*. *guilliermondii*, *Aureobasidium* including *Aureobasidium pullulans*, *Erysiphe* including *E*. *necator*, *Aspergillus*, *Stemphylium*, *Penicillium* including *Penicillium spinulosum*, *P*. *bilaiae*, and *P*. *canescens*, *Alternaria* including *Alternaria alternata* and *A*. *metachromatica*, *Cladosporium* including *C*. *tenuissimum* and *Mycosphaerella* including *M*. *tassiana* (Figures 6A,D). During fermentation, the genera *Hanseniaspora*, including the species *H*. *nectarophila* and *H*. *guilliermondii* and *Aureobasidium*, including the species *A*. *pullulans*, remained the most dominant taxa (Figures 6B,E). Most samples from both varieties demonstrated an increase in the relative abundance of the genus *Saccharomyces*, for the species *S*. *cerevisiae* and *Saccharomyces paradoxus*. Post-fermentation, *H. nectarophila* remained the predominant species in most samples, followed by the genus *Saccharomyces*, with representative species the *S. cerevisiae*, *H. guilliermondii*, and *A. pullulans* (Figures 6C,F). Interestingly, some samples from Kyperounta were characterized by an increase in the relative abundance of the species *Schwanniomyces occidentalis*, were some from Kathikas, Panayia, and Statos of the species *Lachancea thermotolerans*. Furthermore, specifically for the *terroir* from Kathikas, for the variety Maratheftiko, an increase in the relative abundance of *Verticillium leptobactrum*, *Malassezia restricta*, *Cladosporium sphaerospermum*, *Meyerozyma guilliermondii*, *Blumeria graminis*, *Candida diddensiae*, and *Debaryomyces prosopidis* was observed.

**FIGURE 7:**
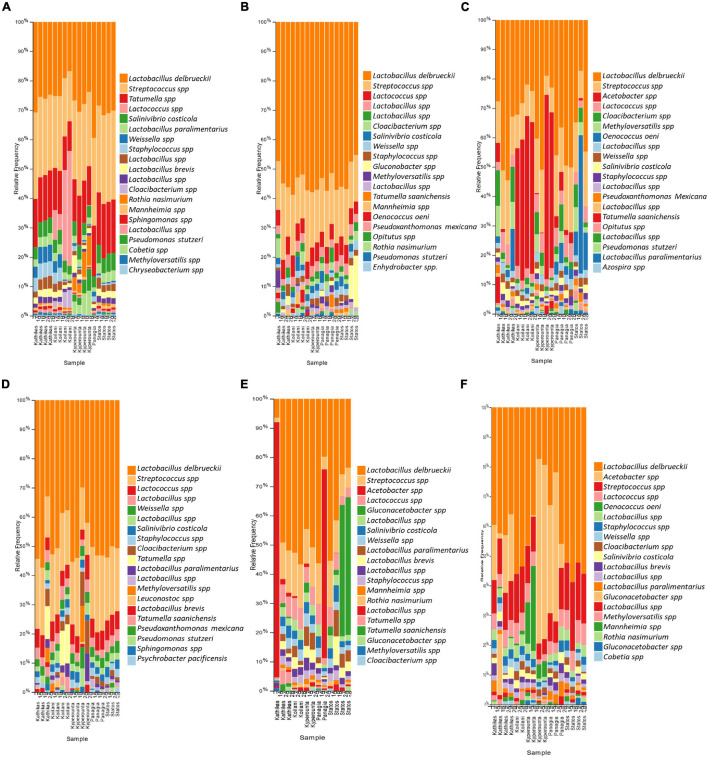
The relative abundance of the twenty most abundant bacteria identified at the species level based on 16S rDNA sequencing for the *terroir* Kathikas, Koilani, Kyperounta, Panayia, and Statos: **(A)** for the variety Xynisteri pre-fermentation; **(B)** for the variety Xynisteri during fermentation; **(C)** for the variety Xynisteri post-fermentation; **(D)** for the variety Maratheftiko pre-fermentation; **(E)** for the variety Maratheftiko during fermentation; **(F)** for the variety Maratheftiko post-fermentation.

The bacterial communities of the Cyprus *terroirs* for both varieties, Xynisteri and Maratheftiko, in grape juice, as revealed by 16S rRNA gene sequencing, were mainly represented by members of the phyla Firmicutes followed by Proteobacteria and in lower abundances of Bacteroides, Actinobacteria, and Verrucomicrobia. *Lactobacillus* species were the most predominant, represented by the species *Lactobacillus delbrueckii*, followed by *Companilactobacillus paralimentarius* and *Levilactobacillus brevis*. Other dominant lactic acid bacteria (LAB) in both grape varieties included the genera *Streptococcus* and *Lactococcus* (Figures 7A,D). Additional detected genera, but in lower relative abundances, included *Tatumella*, represented by the species *T*. *saanichensis*, *Salinivibrio*, represented by the species *S*. *costicola*, *Staphylococcus*, *Cloacibacterium*, and *Rothia*, represented by the species *R*. *nasimurium*, *Mannheimia*, and *Sphingomonas Pseudomonas*, represented by the species *P*. *stutzeri* and *P*. *mexicana*, *Weissella*, *Methyloversatilis*, and *Leuconostoc*. During fermentation, *Lactobacillus*, *Streptococcus*, and *Lactococcus* were the predominant among the bacterial communities for both varieties (Figures 7B,E). Some samples were characterized by an increase in the relative abundance of the genus *Gluconobacter*. Also, for Xynisteri, some samples were characterized by an increase in the relative abundance of the species *O. oeni*, whereas for the variety Maratheftiko of the genus *Acetobacter*. Post-fermentation, apart from the species *L*. *delbrueckii* and the genera *Streptococcus* and *Lactococcus*, an increase in the relative abundance on the genus *Acetobacter* was observed, both for Xynisteri and Maratheftiko (Figures 7C,F). Also, some samples showed a further increase in the relative representation of *O. oeni*.

Comparison of the microbial diversity between pre-fermentation samples obtained from the same vineyard indicated differences in the relative representation of microbes. For instance, for the variety Xynisteri, the relative representation of *A. pullulans* was different between samples from the same vineyard, for both Kathikas (5 and 10%) and Panayia *terroir* (15 and 29%). Similarly, differences were observed in the relative representation of the genus *Aspergillus* for Panayia (13 and 22%) and Statos *terroir* (19 and 39%). Additionally, some differences in the relative representation of microbial species were detected for samples obtained for neighbor vineyards of the same *terroir*. For example, for both varieties, the relative representation of *Penicillium_spinulosum* for Kathikas *terroir* was between 13 and 18% in samples from the one vineyard and about 0–1% for samples from the neighbor vineyard. Similarly, *Erysiphe_necator* indicated a relative representation of about 23% for samples from the one vineyard of Kathikas *terroir* and around 0.05% for samples from the neighbor vineyard. Similar results were observed for *Erysiphe_necator* for Panayia and Statos *terroir*. Also, for the variety Maratheftiko, for Koilani *terroir*, the relative representation of *Cladosporium_tenuissimum* and *Alternaria_alternata* was about 0.4% for samples obtained from one vineyard and about 11 and 31%, respectively, for samples from the neighbor vineyard.

Comparison of the microbial diversity of post-fermentation samples obtained from the same *terroir* also indicated differences in the relative microbial representation. Specifically, for the variety Xynisteri in Kathikas *terroir*, the relative representation of *Saccharomyces* was about 48% in samples obtained from one vineyard and about 4% in samples obtained from the neighbor vineyard. Similarly, for *H. guilliermondii*, the relative representation was about 26% in the one vineyard and about 86% in the neighbor vineyard. Moreover, for the variety Maratheftiko in Kyperounta *terroir*, the relative representation of *Lactobacillus delbrueckii* was about 15% in samples from the one vineyard, and about 26% from samples from the neighbor vineyard. Additionally, differences were observed between samples obtained from the same vineyard. For instance, the relative representation of *A._pullulans* and *Blumeria_graminis* in Koilani *terroir* for the variety Maratheftiko was 3 and 1%, respectively, in one sample and 30% for both species in the other sample.

## Identification of Microbial Biomarkers in Xynisteri and Maratheftiko

The LEfSe algorithm was applied to analyze the identified relative abundances of microbial taxa and determine whether their values were differentially distributed among the five analyzed geographic regions. The analysis was performed from class until species level. The statistical comparisons were executed separately: (i) for the fungal and bacterial diversity; (ii) for the three stages of fermentation: pre- during and post-fermentation; and (iii) for the varieties Xynisteri and Maratheftiko.

### Fungal Biomarkers Associated With the Five *terroirs*

**FIGURE 8:**
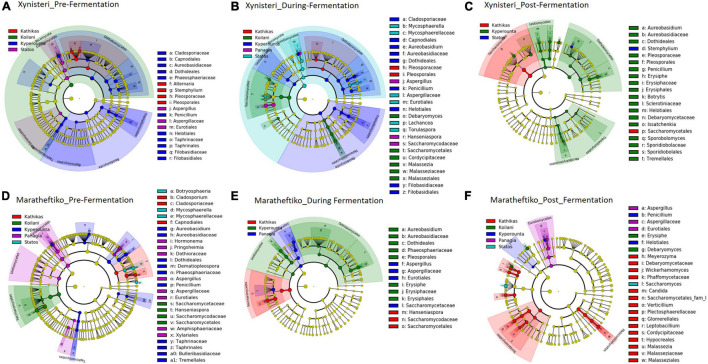
LEfSe analyses of taxon abundances of over-represented fungal taxa among grape wine varieties and among stages of fermentation. **(A)** Variety Xynisteri pre-fermentation; **(B)** variety Xynisteri during fermentation; **(C)** variety Xynisteri post-fermentation; **(D)** variety Maratheftiko pre-fermentation; **(E)** variety Maratheftiko during fermentation; **(F)** variety Maratheftiko post-fermentation.

Pre-fermentation, for the variety Xynisteri, the Kyperounta *terroir* indicated significantly a higher relative representation for the genus *Penicillium* and the species *A. pullulans* (Figure 8A and Table 1). The genus *Stemphylium* and the species *A. alternata* were associated with Kathikas *terroir*. Also, the species *P. spinulosum* indicated a higher relative representation in the Paphos PGI region *terroirs*. For the variety Maratheftiko, in agreement with Xynisteri, the Kyperounta *terroir* had a significantly higher representation of the species *A. pullulans* (Figure 8D and Table 1). In addition, *Cladosporium tennuisium* was associated with Kathikas *terroir* and *Mycosphaerella tassiana* and *Penicillium canescent* with Statos *terroir*. Finally, Koilani *terroir* had a significantly higher representation of the species *H. nectarophila*.

**TABLE 1:** Fungal taxa with significantly higher relative representation in a specific *terroir* (highlighted with red) for the two grape wine varieties during the process of fermentation.

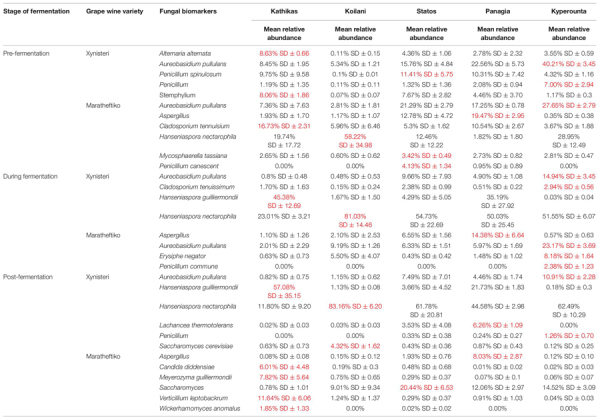

During fermentation, for the variety Xynisteri, in agreement with pre-fermentation, Kyperounta *terroir* indicated a significantly higher representation of *A. pullulans* (Figure 8B and Table 1). In addition, the species *Cladosporium tenuissimum* was associated with Kyperounta. Kathikas had a higher relative representation of *S. paradoxus* and *H. guilliermondii*. Finally, Koilani was correlated with the species *H. nectarophila*. For the variety Maratheftiko, Kyperounta was also associated with *A. pullulans* (Figure 8E and Table 1). In addition, it was associated with the species *Penicillium commune* and *E. necator*. Finally, in agreement with pre-fermentation, Panayia was correlated with *Aspergillus*.

Post-fermentation analysis revealed that the species *A. pullulans* were a biomarker of Kyperounta *terroir* throughout the evolution of the fermentation, especially for the variety Xynisteri. Additionally, some taxa that were associated with specific *terroir* during fermentation indicated also significantly higher representation post-fermentation. These included, for the variety Xynisteri *H. guilliermondii* for the Kathikas and *H. nectarophila* for the Koilani *terroir* (Figure 8C), and for the variety Maratheftiko, *Aspergillus* for the Panayia *terroir* (Figure 8F). Furthermore, some new associations were detected. These included, for the variety Xynisteri Hanseniaspora guilliermondii for the Kathikas and Hanseniaspora nectarophila for the Koilani terroir (Figure 8C) and for the variety Maratheftiko, Aspergillus for the Panayia terroir (Figure 8F). For instance, for the variety Xynisteri, the species *L. thermotolerans* was correlated with Panayia and the species *S. cerevisiae* with Koilani *terroir*. In addition, the species *Meyerozyma guilliermondii*, *Wickerhamomyces anomalus*, *Verticillium leptobackrum*, and *Candida diddensiae* were associated with Kathikas *terroir* and *Saccharomyces* with the Statos *terroir*.

### Bacterial Biomarkers Associated With the Five *terroirs*

**FIGURE 9:**
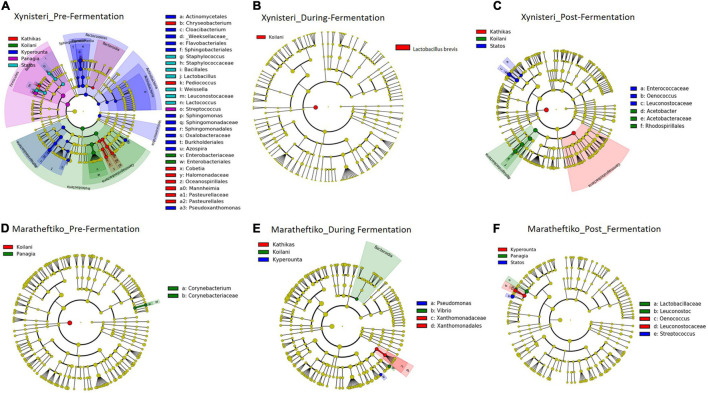
LEfSe analyses of taxon abundances of over-represented bacterial taxa among grape wine varieties and among stages of fermentation. **(A)** Variety Xynisteri pre-fermentation; **(B)** variety Xynisteri during fermentation; **(C)** variety Xynisteri post-fermentation; **(D)** variety Maratheftiko pre-fermentation; **(E)** variety Maratheftiko during fermentation; **(F)** variety Maratheftiko post-fermentation.

Pre-fermentation, for the variety Xynisteri, in Kyperounta *terroir*, the genera *Cloacibacterium*, *Sphingomonas*, *Azospira* and the species *Pseudoxanthomonas mexicana* were significantly more abundant compare to the other *terroirs* (Figure 9A and Table 2). Also, Statos *terroir* had a significantly higher representation of the genera *Weissella* and *Lactococcus* and the species *L*. *delbrueckii* (Figure 9A and Table 2). Post-fermentation, for the variety Xynisteri, the genus *Acetobacter* was associated with Koilani *terroir* (Figure 9C and Table 2). Additionally, for the variety Maratheftiko, Panayia had higher relative representation of *L*. *brevis* and *C*. *paralimentarius* and Kyperounta of *O. oeni* (Figure 9F and Table 2). No significant associations were detected Maratheftiko pre-fermentation (Figure 9D) and for Xynisteri (Figure 9B) and Maratheftiko (Figure 9E) during fermentation.

**TABLE 2:** Bacterial taxa with significantly higher relative representation in a specific *terroir* (highlighted with red) for the two grape wine varieties during the process of fermentation.

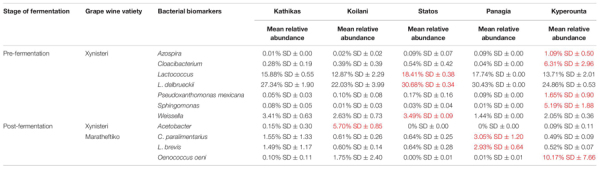

### Microbial Biomarkers Associated With the Grape Wine Varieties Xynisteri and Maratheftiko

Finally, we investigated the presence of microbial biomarkers associated with the varieties Xynisteri and Maratheftiko, in the relative abundance of more than 1%. The analysis revealed that none of the analyzed fungal taxa were correlated with either Xynisteri or Maratheftiko during the process of fermentation (pre-, during, and post-fermentation). Regarding bacteria, the detected differences were during fermentation. Specifically, Xynisteri had a higher relative representation of the LAB *Lactococcus*, *Weissella*, and the species *L*. *delbrueckii*, whereas Maratheftiko with *Gluconacetobacter*, *L*. *brevis*, and *C*. *paralimentarius* (Table 3).

**TABLE 3:** Bacterial taxa with significantly higher relative representation in Xynisteri or Maratheftiko grape wine variety (highlighted with red) during the process of fermentation.

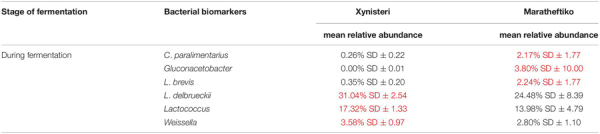

## Discussion

Substantial evidence suggests that grape−associated distinguishable microbial patterns (microbial *terroir*) contribute to the regional identity of the produced wine (Pinto et al., 2015; Bokulich et al., 2016; Anagnostopoulos et al., 2019; Gobbi et al., 2020). The present study was conducted to establish an association between microbial diversity and the different Cyprus wine regions (*terroirs*) during spontaneous wine fermentation of the grape wine varieties Xynisteri and Maratheftiko. To demonstrate this, we applied HTS for the primary in-depth characterization of Cyprus wines microbial diversity, mapping the dissimilarity in the microbial community of five grape wine *terroirs*. The study revealed a distinction in alpha and beta diversity among the five *terroirs*, among the different stages of fermentation and between the varieties Xynisteri and Maratheftiko. Additionally, we identified microbial biomarkers associated with each *terroir* among the different stages of fermentation and for the different wine grape varieties.

The process of AF contributed to a progressive reduction in the fungal alpha diversity, both for Xynisteri and Maratheftiko grape wine varieties. This observation is in agreement with a recent study comparing the fungal diversity of typical *Vitis vinifera* L. var. Sauvignon blanc from Chile between pre-fermented and fermented musts (Mandakovic et al., 2020) and a metataxonomic study of Vino Santo mycobiota in the Italian Alps (Stefanini et al., 2016). During AF, yeast species such as *S*. *cerevisiae* ferment sugars producing ethanol (Stefanini et al., 2016). The stressful environment for some yeast species created by the high alcohol leads to their elimination (Stefanini and Cavalieri, 2018). Therefore, post-fermentation, the microbial consortia are comprised of specific microbial species (Campisano et al., 2014; Bokulich et al., 2016). Apart from AF, MLF (conversion of malic acid into lactic acid) is conducted, generally by LAB (Renouf et al., 2005). Based on our results, the bacterial alpha diversity that comprised the two grape wine varieties remained stable during the process. Pre-fermentation, the areas with the lowest altitude, including Kathikas and Koilani, indicated significantly higher bacterial alpha diversity than Kyperounta, the area with the highest altitude. High altitude environments select specific microbes able to adapt and maintain their metabolic activities in low temperature, high UV irradiation, reduced atmospheric pressure and limited soil nutrients (Kumar et al., 2019). Therefore, the selective pressure of the stressful highest altitude ecosystem of Kyperounta may be responsible for the lower bacterial diversity compared to the other *terroirs*. However, for the variety Xynisteri, the fungal diversity of Kyperounta was significantly higher compared to Koilani, indicating that additional factors might affect these *terroirs* fungal communities’ composition, for instance, cooler climatic conditions and rainfall (Bokulich et al., 2013; Grangeteau et al., 2017).

The Bray-Curtis dissimilarity distance metric defined that the microbial community contributes to distinguishing musts originated from different *terroirs* and grape varieties. This is in line with additional studies based on HTS, revealing microbiological patterns associated with viticulture areas (Bokulich et al., 2012; Pinto et al., 2015; Burns et al., 2016; Portillo Mdel et al., 2016; Mezzasalma et al., 2017). Taylor et al. (2014) identified a distinct difference in the fungal community composition of Central Otago compare to the other four main regions in New Zealand. The distinction we observed was grape wine variety depended. Specifically, the fungal beta diversity of the variety Xynisteri was significantly different among almost all the different *terroirs* from pre- until post-fermentation. For the variety Maratheftiko, the fungal beta diversity of Kyperounta was significantly different from the other *terroir* pre-, during and post-fermentation. Kyperounta was also distinguished from the other *terroir* based on the bacterial diversity, in the pre-fermentation stage, for the variety Xynisteri. In addition, PERMANOVA analysis revealed significant differences in the microbial beta diversity between Xynisteri and Maratheftiko for the stages pre- and post-fermentation. Bokulich et al. (2016) also demonstrated that the distinction of the different viticultural areas and individual vineyards across California depends on the wine grape variety, with Chardonnay showing more powerful microbial composition separation ability than Cabernet Sauvignon.

Pre-fermentation, the fungal diversity was dominated by non-*Saccharomyces* yeasts. These included both for Xynisteri and Maratheftiko the genera *Hanseniaspora*, represented by the species *H*. *nectarophila* and *H*. *guilliermondii*, *Aureobasidium*, including *A*. *pullulans*, *Erysiphe* represented by *E*. *necator*, *Aspergillus*, *Stemphylium*, and *Penicillium* including *P*. *spinulosum*, *P*. *bilaiae*, and *P*. *canescens*, *Alternaria* including *A*. *alternata* and *A*. *metachromatica*, *Cladosporium* including *C*. *tenuissimum* and *Mycosphaerella* represented by *M*. *tassiana*. Similarly, pre-fermentation samples from different wine appellations in Portugal were characterized by a higher relative representation of *A*. *pullulans* and *Hanseniaspora*, but *H*. *uvarum* instead of *H*. *nectarophila* and *H*. *guilliermondii* that we detected (Pinto et al., 2015). The analyzed Cyprus *terroir* fungal diversity of Xynisteri and Maratheftiko differed from the Italian grape varieties Greco and Aglianico analyzed by De Filippis et al. (2017). Specifically, the abundant species they discovered included *Hanseniaspora osmophila*, *Hanseniaspora uvarum*, *Candida stellata*, *Pichia kluyveri*, *Metschnikowia pulcherrima*, *Zygosaccharomyces rouxii*, *Issatchenkia terricola*, *Pichia occidentalis*, *Botrytis cinerea*, *Cladosporium bruhnei*, and *Aspergillus niger*. In agreement with our findings, the genus *Hanseniaspora* was the predominant pre-fermentation for the variety Grenache from Catalonia, Spain (Portillo Mdel and Mas, 2016). Several common genera were detected in an HTS study of unfermented must of the variety *V. vinifera* L. var. Sauvignon blanc in Chile, in which a commercial starter culture of *S. cerevisiae* was added (Mandakovic et al., 2020). Apart from the genus *Saccharomyces* the genera *Hanseniaspora*, *Torularospora*, *Botrytis*, *Penicillium*, *Cladosporium*, *Candida*, and *Aureobasidium* were the most dominant in agreement with most of our findings. The genera *Aureobasidium*, *Penicillium*, *Erysisphe Aspergillus*, and *Alternaria*, were among the predominant genera in an amplicon sequencing study performed in grape juice of the variety Cabernet sauvignon in China (Wei et al., 2018).

The bacterial diversity of pre-fermented samples for Xynisteri and Maratheftiko was dominated by LAB, mostly species of the genera *Lactobacillus* including *L*. *delbrueckii*, followed by *C*. *paralimentarius* and *L*. *brevis*, *Streptococcus* and *Lactococcus*. The genera *Tatumella*, *Salinivibrio*, *Staphylococcus*, *Cloacibacterium*, *Rothia*, *Mannheimia*, *Sphingomonas Pseudomonas*, *Weissella*, *Methyloversatilis*, and *Leuconostoc* were detected in lower relative abundances. In contrast to our results, an HTS study of the wine microbiota from six wine appellations in Portugal revealed that the grape juice was dominated by Proteobacteria members of the families *Enterobacteriaceae* and *Pseudomonadaceae* (Pinto et al., 2015). An HTS longitudinal analysis of about 200 commercial wines during fermentation in California indicated that the dominant taxa pre-fermentation were apart from *Enterobacteriaceae* and *Pseudomonas*, *Leuconostocaceae*, *Bacillaceae*, *Sphingomonas Gluconobacter*, and *Lactobacillus* (Bokulich et al., 2016).

Most of the identified pre-fermentation microbiota was comprised of environmental microorganisms detected in the vineyard ecosystem. Specifically, *A*. *pullulans*, *Aspergillus*, *Penicillium*, *E. necator*, *Alternaria*, *Stemphylium*, *Lactobacillus*, *Streptococcus*, *Staphylococcus*, and *Gluconobacter* were found to be associated with vineyard environment, including soil, grape surface and leaves (Bokulich et al., 2013; Pinto et al., 2014; Zarraonaindia et al., 2015; Wei et al., 2018). Additionally, *Sphingomonas* and *Pseudomonas* species were detected in aboveground plant areas (Whipps et al., 2008; Innerebner et al., 2011). Some species, such as *Pseudomonas* and *Staphylococcus*, were identified as endophytic bacteria (Compant et al., 2011). *Hanseniaspora*, the most dominant genus, is commonly found on grapes (Varela and Borneman, 2017). Species of the *Hanseniaspora* contribute significantly to wine fermentation by controlling the metabolic activity of *S. cerevisiae* and producing metabolites that affect the wine color and other sensorial characteristics (Martin et al., 2018). *Hanseniaspora nectarophila*, the most dominant species in most pre-fermentation samples, was first isolated from ephemeral flowers and its role in wine fermentation remains undetermined (Čadež et al., 2014). Mezzasalma et al. (2018) identified geographical signatures in the grape microbiome associated with its origin. The researchers found that the vineyard soil microbiome was about 60% common with the grape microbiome. Through a machine learning system, they predicted the origin of the grape based on the microbiota. Gobbi et al. (2020) analyzed the soil microbiota of 200 vineyards from four continents by using an amplicon sequencing approach. Even though we analyzed grape juice and not soil samples, their results highlight the unique microbiome found in the Cyprus vineyards. Specifically, countries such as Italy, Croatia, Argentina, Chile, and South Africa had a higher relative abundance of *Solicoccozyma*, whereas Portugal, Germany, Australia, Denmark, and South Africa of *Fusarium*. Additional dominant species that Gobbi et al. detected include *Gibberella intricans* in Spain, Portugal, and Denmark, *Tausonia pullulans* in Hungary and Spain, *Metarhizium_robertsii* in Denmark, *Sclerotinia sclerotiorum* and *Rhizopus arrhizus* in France, and *Truncatella* in Germany. Some species that we also detected in our study included *Cladosporium*, which was dominant in Portugal, Germany, Australia, and Denmark, *Alternaria* in Chile and *Aspergillus* in South Africa.

Generally, AF is performed by the contribution of several microbes, with *Saccharomyces* eventually governing (Fleet, 2008). However, spontaneous AF may be driven by non-Saccharomyces yeasts, something unwanted by the wine industries because it can affect the wine’s aromatic quality (Zott et al., 2011; Liu et al., 2016). Evaluation of microbial community dynamics during and post-spontaneous AF revealed that the process in most Cyprus must samples was driven by non-*Saccharomyces* yeasts, including *H*. *nectarophila and H*. *guilliermondii*. However, an increase in the relative abundance of *Saccharomyces* and the species *S*. *cerevisiae* and *S*. *paradoxus* was indicated during fermentation, and *Saccharomyces* became abundant by the end of fermentation in only a few samples. In agreement, Stefanini et al. (2016) observed that during Vino Santo must fermentation in Italy, in one of the three tested wineries, the mycobiota was dominated by *H. osmophila*, and *S*. *cerevisiae* was detected in lower relative abundances. In the other two wineries, though, the AF was driven by *S*. *cerevisiae*. Our findings suggest that the vineyard microbiota guides the process of fermentation since species such as *H. nectarophila*, *H. guilliermondii* and *A. pullulans* remained among the predominant species from pre- until post-fermentations. Noteworthy, Čadež et al. (2014) reported that the discrimination of *H. nectarophila* from the other closely related species, including *H. guilliermondii Hanseniaspora meyeri*, *Hanseniaspora opuntiae*, and *Hanseniaspora clermontiae*, was based not only on the ITS and D1/D2 LSU sequences similarity comparison but also the sequences of the gene coding for actin. This is because the differences with the aforementioned species regarding the D1/D2 LSU and ITS sequences were only were four nucleotides, which were not enough to separate *H. nectarophila* into separate species. Other species, such as *A. alternata*, *E. necator*, *P. spinulosum* continued to be among the low abundant species (>1%) from the pre- until post-fermentations. These results are in agreement with other metataxonomic studies (Bokulich et al., 2013, 2016; Morrison-Whittle and Goddard, 2018).

Several factors affect the dominance of *S. cerevisiae* during AF. The increased tolerance of *Saccharomyces* species to ethanol and its fast growth rate is the primary factor contributing to its dominance. However, apart from *Saccharomyces* species, alcohol tolerant non-*Saccharomyces* yeasts may drive AF. For instance, Sirén et al. (2019) identified *Hanseniaspora* as the predominant species in post-fermentation Riesling must following spontaneous *in vitro* fermentation. The tolerance of *Hanseniaspora* to ethanol was found to increase by low temperature (10–15°C) (Erten, 2002). Still, the fermentation of Cyprus musts was performed at 18°C, indicating that the temperature might not be associated with *Hanseniaspora’s* dominance. Boynton and Greig (2016) applied HTS to indicate that the ability of *Saccharomyces* to lead the process of fermentation in must samples is prevented by increased species richness, which might be an affecting factor for *Saccharomyces* suppression in our study. Another affecting factor could be the availability of sugars since the metabolic activity of *S*. *cerevisiae* is induced by high sugar concentrations (Lleixà et al., 2016). The sugar concentration of both Xynisteri and Maratheftiko is >20 Brix, which is considered high. Furthermore, other studies supported that some *S*. *cerevisiae* strains secrete antimicrobial peptides that prevent the growth of non-*Saccharomyces* yeasts (Rai and Jeyaram, 2015; Fakruddin et al., 2017). For instance, the isolated strain *S*. *cerevisiae* CCMI 885 secreted small peptides with antimicrobial activity against *H*. *guilliermondii* (Albergaria et al., 2010). However, some *A*. *pullulans* strains were found to produce aureobasidin A, which show a powerful fungicidal action against *S*. *cerevisiae* and *Candida* species (Heidler and Radding, 1995; Zhong et al., 2000). *A*. *pullulans* were found to be among the predominant species in some of our samples.

The amplicon sequencing analysis indicated that the most abundant species during MLF was *L*. *delbrueckii*. The metabolic activity of LAB leads to deacidification of the must by converting malic acid into lactic acid (Lonvaud-Funel, 1999). Apart from the LAB *Lactobacillus*, *Streptococcus* and *Lactococcus*, some during fermentation samples were characterized by an increase in the relative abundance of the genera *Gluconobacter* and *Acetobacter* and some post-fermentation samples by the dominance of *Acetobacter*. The metabolic activity of acetic acid bacteria may negatively impact the quality of the produced wine due to the production of acetic acid (Zoecklein et al., 2000). Bokulich et al. (2016) revealed that increased bacterial richness prevents AF and identified antagonistic relationships between *Lactobacillus* spp., *Gluconobacter* and *H*. *uvarum*, with *S*. *cerevisiae*, competing for nutrients availability. In agreement, Berbegal et al. (2019) suggested that *Saccharomyces* exerts selective pressure on the spoilage *Acetobacter* and *Gluconobacter* and promotes the dominance of *Oenococcus*. In the present study, only one sample was characterized by the dominance of *O. oeni*. Based on these data, the supremacy of *Hanseniaspora* and the suppression of *Saccharomyces* may be responsible for the suppression of growth of *Oenococcus*, favoring the increase in the relative abundance of acetic acid bacteria that we observed.

According to a recent review article by Griggs et al. (2021), the wine-relevant microbial diversity of the vineyard affects the outcome of the fermentation and the quality of the produced wine. In our study, we observed differences in the relative representation of microbes between samples from the same vineyard and neighbor vineyards. These differences affected the process of fermentation, leading to alterations in the relative representation of post-fermentation microbial species. Specifically, for the variety Xynisteri, samples obtained from one vineyard of Kathikas *terroir* indicated a higher relative representation of *Saccharomyces*, whereas samples obtained from the neighbor vineyard of *H. guilliermondii*. As described above, species of the genera *Saccharomyces* and *Hanseniaspora* are drivers of AF. However, as described by Griggs et al., our knowledge regarding the factors affecting the microbial biodiversity shaping among vineyards and drive the process of fermentation remains limited, especially regarding spontaneous fermentations. Noteworthy, all fermentations were performed in the same place, under the same conditions. These findings provide additional evidence that the vineyard microbiota influences the microbial community shaping and the drivers of fermentation.

Finally, the study revealed associations among the microbial diversity and the different *terroirs* and wine varieties, from pre- until post-fermentation. Comparison of the fungal diversity among the five different *terroirs* and the three different stages of fermentation revealed that the species *A*. *pullulans* were associated with the Kyperounta *terroir*, both for Xynisteri and Maratheftiko, pre-, during, and post-fermentation. *A*. *pullulans* are among the dominant microbiota of grape juice (Pinto et al., 2015; Sternes et al., 2017) and considered anti-phytopathogen microorganism, expressing anti-fungal and anti-bacterial activity against several important post-harvest pathogens such as *Botrytis* and *Bacillus* (Sharma et al., 2009; Grube et al., 2011; Prasongsuk et al., 2018). Additionally, these species have an essential contribution to wine’s color, sensorial characteristics and clarification efficiency (Merín and Morata de Ambrosini, 2018; Onetto et al., 2020). Apart from *A*. *pullulans*, *Penicillium* was also associated with the Kyperounta *terroir*, both for Xynisteri and Maratheftiko, pre-, during, and post-fermentation. This genus has an important contribution to the wine industry since it may change grape wines’ chemical composition affecting the microbial community shaping during fermentation (Nigam et al., 2017). As a result, it affects the colors and flavors of the produced wine. Kathikas *terroir* indicated a significantly higher representation of the species *A*. *alternata* pre- and during fermentation for the variety Xynisteri. *A*. *alternata* is an important contaminant in grape juice. It produces toxic metabolites, the accumulation of which during winemaking is risky for consumers health (Prendes et al., 2021). Statos *terroir* was distinguished by the psychrophilic, acidotolerant species *P. spinulosum* and the antibiotic canescin producing species *P. canescent* for the variety Xynisteri and Maratheftiko, respectively, during pre-fermentation. Koilani *terroir* was characterized by a significantly higher representation of *H. nectarophila* pre-fermentation for the variety Maratheftiko and during and post-fermentation for the variety Xynisteri. Furthermore, the study revealed bacterial biomarkers associated with the five grape wine *terroirs*. However, none of the identified bacterial biomarkers was detected from the beginning until the end of the fermentation process.

Since the aim of our study is to provide evidence of the existence of different microbial patterns among the five terroirs, it is important to mention that Kokkinofta et al. (2017) identified differences in the five terroir that we analyzed in the present study, for the same wine varieties (Xynisteri and Maratheftiko) by applying SNIF-NMR, IRMS and inductively coupled plasma atomic emission spectroscopy (ICP-AES). Specifically, chemometrics analysis of elements and isotopes revealed differentiation between the varieties Maratheftiko and Xynisteri, and a significant distinction between the Pafos PGI region and the Lemessos PGI region and among the wineries Kamanterena and Vassilikon (PDO Laona Akamas) in the Pafos PGI region and Pelendri (PDO Pitsilia) and Koilani village (PDO Krasochoria), Lemessos PGI region. Furthermore, we need to mention that our analysis was restricted to only four samples per *terroir* and to only 1 year of sampling, which is a limiting factor, considering that regional environmental factors, such as climatic conditions, may affect the microbial community composition. Also, our analysis included multiple comparisons, which increase the possibility of significant differences to be discovered by chance. Although in most of the comparisons the adjusted values remained significant, in a few of them didn’t, indicating a limitation associated with multiple comparisons performance.

## Conclusion

The present metataxonomic study is the first attempt to distinguish the five different Cyprus wine *terroirs’* microbial diversity for the two grape wine varieties Xynisteri and Maratheftiko during the stages of pre- until post-spontaneous fermentation. The study revealed a significant reduction in the fungal diversity during the stages of fermentation and in the bacterial diversity between the areas with the lowest altitude, compared to the area with the highest altitude pre-fermentation. Most importantly, the study defined that microbial diversity is a key factor for distinguishing grape wines from different *terroirs* and grape wine varieties. In the future, apart from the present, more grape wine varieties andsamples from multiple harvests are to be analyzed using an amplicon sequencing approach. This will allow us to compare the alterations in the microbial diversity throughout different years. The metataxonomic analysis may be combined with predictive functional analysis to reveal the influence of the bacterial and fungal community associated metabolic pathways in the wine aromatic profile and flavors. Also, it may be combined with SNIF-NMR, IRMS and inductively coupled plasma atomic emission spectroscopy (ICP-AES), as applied by Kokkinofta et al. (2017), and DNA fingerprint characterization studies. These methodologies may assist to differentiate authentic wines from possible fraud products that may exist in the market and provide a fingerprint for defining the authenticity of Cyprus wines. The present study’s findings will further assist in the improvement of the qualitative characteristics of Cyprus wines.

## Data Availability Statement

Our data are publicly available at the following link: https://www.ncbi.nlm.nih.gov/sra/?term=PRJNA731461.

## Author Contributions

DT and MM: conceptualization and writing–review and editing. MM and CK: grape wine fermentation. EK: methodology, formal analysis, investigation, data curation, and writing–original draft preparation. DT: resources, supervision, project administration, and funding acquisition. All authors have read and agreed to the published version of the manuscript.

## Conflict of Interest

CK is employed by Kyperounta Winery. The remaining authors declare that the research was conducted in the absence of any commercial or financial relationships that could be construed as a potential conflict of interest.

## Publisher’s Note

All claims expressed in this article are solely those of the authors and do not necessarily represent those of their affiliated organizations, or those of the publisher, the editors and the reviewers. Any product that may be evaluated in this article, or claim that may be made by its manufacturer, is not guaranteed or endorsed by the publisher.

## Footnotes

1
https://support.illumina.com/content/dam/illumina-support/documents/documentation/chemistry_documentation/metagenomic/fungal-metataxonomic-demonstrated-protocol-1000000064940-01.pdf
