# Supplementary material for: Metataxonomic Analysis of Grape Microbiota During Wine Fermentation Reveals the Distinction of Cyprus Regional terroirs
Source: Front Microbiol. 2021 Sep 22;12:726483. doi: 10.3389/fmicb.2021.726483 (PMC8494061; doi:10.3389/fmicb.2021.726483)
Supplement: Supplementary file 1 [file Data_Sheet_1.docx]

Supplementary Material

## Supplementary Tables

Table S1. Information about samples’ sugar concentration, yeast assimilable nitrogen, pH, total acidity

| Terroir/Parameter | Grape Variety | Sugar concentration | Yeast assimilable nitrogen | pH | Total Acidity |
| --- | --- | --- | --- | --- | --- |
| Kathikas1A | Xynisteri | 204.2 g/l | 160 mg/l | 3.37 | 2.64 g/l σε H2SO4 |
| Kathikas1B |  | 204.1 g/l | 160 mg/l | 3.31 | 2.63 g/l σε H2SO4 |
| Kathikas2A |  | 204.3 g/l | 160 mg/l | 3.4 | 2.64 g/l σε H2SO4 |
| Kathikas2B |  | 204.1 g/l | 160 mg/l | 3.42 | 2.64 g/l σε H2SO4 |
| Koilani1A |  | 222.9 g/l | 170 mg/l | 3.42 | 2.84 g/l σε Η2SO4 |
| Koilani1B |  | 223.2 g/l | 170 mg/l | 3.31 | 2.84 g/l σε Η2SO4 |
| Koilani2A |  | 223.1 g/l | 170 mg/l | 3.44 | 2.80 g/l σε Η2SO4 |
| Koilani2B |  | 223.2 g/l | 170 mg/l | 3.43 | 2.81 g/l σε Η2SO4 |
| Panayia1A |  | 205.1 g/l | 160 mg/l | 3.28 | 2.63 g/l σε H2SO4 |
| Panayia1B |  | 205.2 g/l | 160 mg/l | 3.24 | 2.65 g/l σε H2SO4 |
| Panayia2A |  | 205.4 g/l | 160 mg/l | 3.22 | 2.64 g/l σε H2SO4 |
| Panayia2B |  | 205.1 g/l | 160 mg/l | 3.32 | 2.63 g/l σε H2SO4 |
| Statos1A |  | 206 g/l | 160 mg/l | 3.31 | 2.91 g/l σε Η2SO4 |
| Statos1B |  | 206.1 g/l | 160 mg/l | 3.33 | 2.91 g/l σε Η2SO4 |
| Statos2A |  | 206 g/l | 160 mg/l | 3.28 | 2.91 g/l σε Η2SO4 |
| Statos2B |  | 206 g/l | 160 mg/l | 3.34 | 2.91 g/l σε Η2SO4 |
| Kyperounta1A |  | 224.2 g/l | 165 mg/l | 3.38 | 2.55 g/l σε H2SO4 |
| Kyperounta1B |  | 224.1 g/l | 165 mg/l | 3.42 | 2.56 g/l σε H2SO4 |
| Kyperounta2A |  | 224.1 g/l | 165 mg/l | 3.35 | 2.62 g/l σε H2SO4 |
| Kyperounta2B |  | 224.1 g/l | 165 mg/l | 3.39 | 2.63 g/l σε H2SO4 |
| Kathikas1A | Maratheftiko | 324.5 g/l | 165 mg/l | 3.41 | 3.21 g/l σε Η2SO4 |
| Kathikas1B |  | 324.7 g/l | 165 mg/l | 3.39 | 3.22 g/l σε Η2SO4 |
| Kathikas2A |  | 324.3 g/l | 165 mg/l | 3.37 | 3.19 g/l σε Η2SO4 |
| Kathikas2B |  | 324.0 g/l | 165 mg/l | 3.39 | 3.21 g/l σε Η2SO4 |
| Koilani1A |  | 230.2 g/l | 175mg/l | 3.1 | 4.23 g/l σε H2SO4 |
| Koilani1B |  | 230.2 g/l | 175mg/l | 3.15 | 4.24 g/l σε H2SO4 |
| Koilani2A |  | 230.2 g/l | 175mg/l | 3.11 | 4.34 g/l σε H2SO4 |
| Koilani2B |  | 230.1 g/l | 175mg/l | 3.13 | 4.32 g/l σε H2SO4 |
| Panayia1A |  | 212.5 g/l | 165 mg/l | 3.26 | 5.58 g/l σε H2SO4 |
| Panayia1B |  | 212.6 g/l | 165 mg/l | 3.23 | 5.59 g/l σε H2SO4 |
| Panayia2A |  | 212.5 g/l | 165 mg/l | 3.25 | 5.59 g/l σε H2SO4 |
| Panayia2B |  | 212.5 g/l | 165 mg/l | 3.28 | 5.59 g/l σε H2SO4 |
| Statos1A |  | 209.2 g/l | 165 mg/l | 3.35 | 4.65 g/l σε H2SO4 |
| Statos1B |  | 209.3 g/l | 165 mg/l | 3.32 | 4.64 g/l σε H2SO4 |
| Statos2A |  | 209.1 g/l | 165 mg/l | 3.28 | 4.67 g/l σε H2SO4 |
| Statos2B |  | 209.1 g/l | 165 mg/l | 3.30 | 4.68 g/l σε H2SO4 |
| Kyperounta1A |  | 240.1 g/l | 180mg/l | 3.20 | 3.51 g/l σε Η2SO4 |
| Kyperounta1B |  | 240.1 g/l | 180mg/l | 3.22 | 3.55 g/l σε Η2SO4 |
| Kyperounta2A |  | 240.2 g/l | 180mg/l | 3.22 | 3.60 g/l σε Η2SO4 |
| Kyperounta2B |  | 240.2 g/l | 180mg/l | 3.21 | 3.63 g/l σε Η2SO4 |

**Table S2**. Sample information, bacterial alpha diversity indexes and observed OTUs for the variety Xynisteri

| Sample ID | Stage of fermentation | Reads passing filter | Denoised reads | Shannon | Simpson | Chao1 | Observed OTUs |
| --- | --- | --- | --- | --- | --- | --- | --- |
| Kathikas1A | Pre-fermentation | 50116 | 44580 | 3.491 | 0.852 | 138 | 129 |
| Kathikas1B |  | 33041 | 29659 | 3.790 | 0.884 | 122 | 116 |
| Kathikas2A |  | 35336 | 23555 | 3.894 | 0.877 | 144 | 138 |
| Kathikas2B |  | 35336 | 31968 | 3.947 | 0.879 | 160 | 154 |
| Koilani1A |  | 41309 | 37063 | 3.708 | 0.862 | 138 | 133 |
| Koilani1B |  | 38998 | 34950 | 3.771 | 0.864 | 139 | 128 |
| Koilani2A |  | 38240 | 33858 | 3.375 | 0.841 | 86 | 82 |
| Koilani2B |  | 38417 | 34210 | 3.214 | 0.808 | 85 | 83 |
| Statos1A |  | 26306 | 23917 | 3.594 | 0.856 | 113 | 112 |
| Statos1B |  | 35604 | 31776 | 3.353 | 0.848 | 106 | 104 |
| Statos2A |  | 40095 | 35798 | 3.415 | 0.857 | 126 | 119 |
| Statos2B |  | 40230 | 36292 | 3.358 | 0.824 | 127 | 119 |
| Panayia 1A |  | 32979 | 28763 | 3.041 | 0.806 | 94 | 89 |
| Kyperounta1A |  | 18010 | 15651 | 3.122 | 0.767 | 85 | 85 |
| Kyperounta1B |  | 20298 | 18000 | 2.800 | 0.698 | 76 | 76 |
| Kyperounta2A |  | 61209 | 54433 | 3.373 | 0.808 | 150 | 128 |
| Kyperounta2B |  | 35819 | 31239 | 2.709 | 0.685 | 93 | 91 |
| Kathikas1A | During Fermentation | 39168 | 34918 | 3.805 | 0.858 | 118 | 108 |
| Kathikas2A |  | 27067 | 24663 | 2.682 | 0.750 | 78 | 76 |
| Kathikas2B |  | 12837 | 11206 | 3.093 | 0.807 | 66 | 66 |
| Koilani1A |  | 37536 | 33898 | 3.618 | 0.795 | 112 | 101 |
| Koilani1B |  | 43601 | 39373 | 3.146 | 0.843 | 162 | 136 |
| Koilani2A |  | 37913 | 34307 | 3.633 | 0.848 | 129 | 121 |
| Koilani2B |  | 48659 | 43738 | 3.276 | 0.843 | 117 | 96 |
| Panayia1A |  | 121337 | 109574 | 3.549 | 0.858 | 78 | 78 |
| Panayia1B |  | 57336 | 51663 | 3.157 | 0.808 | 76 | 76 |
| Panayia2A |  | 121337 | 79687 | 4.036 | 0.879 | 226 | 152 |
| Panayia2B |  | 57336 | 38100 | 3.590 | 0.847 | 176 | 138 |
| Statos1A |  | 56152 | 50502 | 3.822 | 0.860 | 165 | 141 |
| Statos1B |  | 37894 | 34331 | 3.551 | 0.836 | 111 | 99 |
| Statos2A |  | 32481 | 29455 | 3.447 | 0.845 | 95 | 90 |
| Statos2B |  | 27496 | 24732 | 3.467 | 0.856 | 87 | 85 |
| Kyperounta1A |  | 62350 | 56770 | 3.419 | 0.833 | 159 | 130 |
| Kyperounta1B |  | 38133 | 34581 | 3.375 | 0.834 | 115 | 97 |
| Kyperounta2A |  | 82731 | 73209 | 2.761 | 0.700 | 123 | 90 |
| Kyperounta2B |  | 16313 | 14469 | 2.623 | 0.692 | 60 | 60 |
| Kathikas1A | Post-fermentation | 65655 | 58488 | 4.169 | 0.905 | 142 | 122 |
| Kathikas1B |  | 78320 | 70756 | 3.574 | 0.834 | 196 | 152 |
| Kathikas2A |  | 35243 | 31735 | 3.025 | 0.782 | 109 | 104 |
| Kathikas2B |  | 49459 | 44787 | 4.048 | 0.898 | 117 | 110 |
| Koilani1A |  | 37502 | 32953 | 3.738 | 0.886 | 107 | 98 |
| Koilani1B |  | 37947 | 33653 | 3.508 | 0.870 | 98 | 96 |
| Koilani2A |  | 64410 | 56446 | 3.724 | 0.889 | 126 | 104 |
| Koilani2B |  | 44803 | 39538 | 3.596 | 0.882 | 100 | 89 |
| Panayia 1A |  | 62166 | 56731 | 3.417 | 0.820 | 150 | 128 |
| Panayia 1B |  | 19981 | 18003 | 3.630 | 0.852 | 88 | 87 |
| Panayia 2A |  | 39681 | 35838 | 3.114 | 0.793 | 107 | 98 |
| Panayia 2B |  | 15838 | 14494 | 3.223 | 0.813 | 64 | 63 |
| Statos1A |  | 34920 | 31243 | 3.767 | 0.868 | 108 | 102 |
| Statos2A |  | 13232 | 11426 | 3.347 | 0.820 | 65 | 65 |
| Statos2B |  | 49022 | 43633 | 3.931 | 0.875 | 209 | 180 |
| Kyperounta1A |  | 48667 | 43423 | 4.121 | 0.883 | 219 | 192 |
| Kyperounta1B |  | 405275 | 358884 | 3.766 | 0.847 | 466 | 234 |
| Kyperounta2A |  | 50745 | 44110 | 3.448 | 0.826 | 130 | 112 |
| Kyperounta2B |  | 55765 | 47548 | 3.489 | 0.845 | 134 | 112 |

**Table S3**. Sample information, fungal alpha diversity indexes and observed OTUs for the variety Xynisteri

| Sample-ID | Stage of fermentation | Reads passing filter | Denoised reads | Shannon | Simpson | Chao1 | Observed OTUs |
| --- | --- | --- | --- | --- | --- | --- | --- |
| Kathikas1A | Pre-fermentation | 26882 | 23841 | 4.160888 | 0.911352 | 111 | 98 |
| Kathikas2A |  | 49893 | 41817 | 4.101491 | 0.916968 | 89 | 71 |
| Kathikas2B |  | 31542 | 28276 | 3.931841 | 0.904463 | 87 | 74 |
| Koilani1A |  | 23703 | 22750 | 2.57427 | 0.758344 | 56 | 48 |
| Koilani1B |  | 22526 | 21344 | 2.829526 | 0.792656 | 68 | 56 |
| Koilani2A |  | 26931 | 24508 | 2.677189 | 0.775904 | 60 | 55 |
| Koilani2B |  | 7892 | 7443 | 2.931498 | 0.809841 | 48 | 47 |
| Panayia 1A |  | 9813 | 9194 | 3.414642 | 0.849575 | 57 | 57 |
| Panayia 1B |  | 10610 | 9714 | 4.145076 | 0.889616 | 98 | 96 |
| Panayia 2A |  | 7954 | 7321 | 4.314411 | 0.916449 | 103 | 102 |
| Statos1A |  | 14131 | 13294 | 3.773636 | 0.867954 | 94 | 85 |
| Statos2A |  | 6221 | 5781 | 4.170752 | 0.918752 | 78 | 78 |
| Kyperounta1A |  | 12698 | 11991 | 5.024418 | 0.949312 | 139 | 127 |
| Kyperounta1B |  | 7633 | 7212 | 4.954032 | 0.947257 | 105 | 104 |
| Kyperounta2A |  | 66446 | 63041 | 5.056766 | 0.948498 | 288 | 175 |
| Kyperounta2B |  | 19376 | 18370 | 4.868649 | 0.943732 | 138 | 123 |
| Kathikas1A | During Fermentation | 11258 | 10390 | 2.806404 | 0.76743 | 50 | 48 |
| Kathikas1B |  | 8442 | 7628 | 2.870982 | 0.762529 | 52 | 51 |
| Kathikas2A |  | 10565 | 9658 | 2.617429 | 0.688068 | 43 | 43 |
| Kathikas2B |  | 21179 | 19327 | 2.340812 | 0.640553 | 37 | 34 |
| Koilani1A |  | 16805 | 5472 | 3.629299 | 0.885603 | 46 | 46 |
| Koilani1B |  | 17234 | 8293 | 2.437027 | 0.721714 | 32 | 32 |
| Koilani2A |  | 15574 | 9767 | 3.356411 | 0.834728 | 69 | 65 |
| Koilani2B |  | 12272 | 10280 | 3.571339 | 0.868279 | 76 | 74 |
| Panayia 1A |  | 24673 | 23238 | 2.623319 | 0.729445 | 59 | 48 |
| Panayia 1B |  | 15104 | 13727 | 2.343758 | 0.697193 | 43 | 40 |
| Panayia 2A |  | 11770 | 11107 | 2.889297 | 0.759945 | 48 | 45 |
| Panayia 2B |  | 6131 | 5738 | 2.554978 | 0.696279 | 32 | 32 |
| Statos1A |  | 27927 | 24608 | 3.689932 | 0.888112 | 64 | 52 |
| Statos1B |  | 34531 | 31948 | 3.531892 | 0.870172 | 63 | 53 |
| Statos2A |  | 10418 | 9533 | 2.392814 | 0.690951 | 43 | 42 |
| Statos2B |  | 17962 | 16234 | 2.442702 | 0.707844 | 51 | 46 |
| Kyperounta1A |  | 21917 | 20581 | 3.665535 | 0.846548 | 102 | 86 |
| Kyperounta1B |  | 23187 | 21651 | 3.818551 | 0.867219 | 110 | 90 |
| Kyperounta2A |  | 15857 | 14851 | 3.999005 | 0.89631 | 69 | 64 |
| Kyperounta2B |  | 20954 | 19964 | 3.501855 | 0.850305 | 66 | 55 |
| Kathikas1A | Post-fermentation | 10487 | 9516 | 2.329715 | 0.696352 | 35 | 35 |
| Kathikas1B |  | 13213 | 11788 | 2.499828 | 0.709493 | 43 | 43 |
| Kathikas2A |  | 25829 | 22112 | 1.151093 | 0.295457 | 39 | 36 |
| Kathikas2B |  | 20563 | 18749 | 0.767565 | 0.175586 | 31 | 31 |
| Koilani1A |  | 12742 | 10801 | 2.10213 | 0.639974 | 47 | 47 |
| Koilani1B |  | 11213 | 10148 | 2.590318 | 0.695335 | 69 | 69 |
| Koilani2A |  | 14973 | 12878 | 2.207121 | 0.654487 | 44 | 44 |
| Koilani2B |  | 17327 | 15273 | 2.213139 | 0.663718 | 50 | 49 |
| Panayia 1A |  | 26729 | 19913 | 2.928402 | 0.823815 | 53 | 52 |
| Panayia 1B |  | 19883 | 14630 | 2.906557 | 0.818924 | 36 | 35 |
| Panayia 2A |  | 14277 | 10111 | 2.894656 | 0.814757 | 35 | 35 |
| Panayia 2B |  | 20258 | 14567 | 2.892361 | 0.81555 | 37 | 35 |
| Statos1A |  | 16149 | 14483 | 3.14589 | 0.836489 | 57 | 56 |
| Statos1B |  | 20666 | 18334 | 2.931345 | 0.820017 | 46 | 45 |
| Statos2A |  | 17036 | 13264 | 2.087717 | 0.662565 | 48 | 47 |
| Statos2B |  | 16720 | 14103 | 1.937239 | 0.644656 | 49 | 48 |
| Kyperounta1A |  | 12990 | 10391 | 2.303812 | 0.70791 | 51 | 51 |
| Kyperounta1B |  | 14930 | 11357 | 2.500297 | 0.744799 | 64 | 64 |
| Kyperounta2A |  | 20580 | 11850 | 3.216376 | 0.856476 | 43 | 43 |
| Kyperounta2B |  | 430689 | 231139 | 3.280609 | 0.861166 | 137 | 84 |

**Table S4**. Sample information, bacterial alpha diversity indexes and observed OTUs for the variety Maratheftiko

| Sample-ID | Stage of fermentation | Reads passing filter | Denoised reads | Shannon | Simpson | Chao1 | Observed OTUs |
| --- | --- | --- | --- | --- | --- | --- | --- |
| Kathikas1A | Pre-fermentation | 58567 | 52143 | 2.998 | 0.792 | 127 | 112 |
| Kathikas1B |  | 13976 | 12473 | 3.023 | 0.800 | 63 | 63 |
| Kathikas2A |  | 41053 | 36896 | 3.580 | 0.844 | 113 | 106 |
| Koilani1A |  | 62744 | 55856 | 3.801 | 0.863 | 149 | 131 |
| Koilani1B |  | 108561 | 97297 | 3.701 | 0.854 | 203 | 148 |
| Koilani2A |  | 37225 | 33216 | 3.477 | 0.845 | 99 | 95 |
| Koilani2B |  | 34095 | 30654 | 3.600 | 0.851 | 111 | 105 |
| Panayia 1A |  | 36879 | 32593 | 3.492 | 0.862 | 110 | 103 |
| Panayia 1B |  | 44435 | 39442 | 3.331 | 0.853 | 105 | 96 |
| Panayia 2A |  | 33795 | 30269 | 3.352 | 0.862 | 106 | 100 |
| Statos1A |  | 49240 | 44692 | 3.310 | 0.849 | 107 | 89 |
| Statos1B |  | 13897 | 12678 | 3.102 | 0.828 | 59 | 59 |
| Statos2A |  | 59743 | 54340 | 3.215 | 0.824 | 131 | 105 |
| Statos2B |  | 39607 | 35706 | 3.427 | 0.851 | 126 | 114 |
| Kyperounta1A |  | 34663 | 30958 | 3.124 | 0.803 | 103 | 95 |
| Kyperounta1B |  | 39109 | 35559 | 2.860 | 0.783 | 83 | 77 |
| Kyperounta2A |  | 42047 | 37585 | 2.751 | 0.729 | 109 | 101 |
| Kyperounta2B |  | 16954 | 15192 | 3.779 | 0.885 | 95 | 94 |
| Kathikas1A | During Fermentation | 35285 | 30128 | 2.329 | 0.620 | 80 | 79 |
| Kathikas2A |  | 40766 | 36340 | 3.708 | 0.858 | 123 | 117 |
| Kathikas2B |  | 34578 | 31103 | 3.303 | 0.817 | 101 | 95 |
| Koilani1A |  | 51387 | 46415 | 3.545 | 0.836 | 125 | 114 |
| Koilani1B |  | 41046 | 37263 | 3.173 | 0.804 | 119 | 109 |
| Panayia 1A |  | 429110 | 386927 | 3.497 | 0.820 | 403 | 206 |
| Panayia 1B |  | 102577 | 90401 | 3.295 | 0.795 | 162 | 120 |
| Panayia 2A |  | 43144 | 36510 | 2.923 | 0.769 | 89 | 84 |
| Statos1A |  | 41375 | 37096 | 3.366 | 0.847 | 108 | 103 |
| Statos1B |  | 15290 | 13757 | 3.695 | 0.872 | 77 | 77 |
| Statos2A |  | 27606 | 24168 | 3.654 | 0.876 | 90 | 86 |
| Statos2B |  | 46280 | 40437 | 3.650 | 0.874 | 113 | 101 |
| Kyperounta1A |  | 31455 | 28488 | 3.666 | 0.874 | 115 | 109 |
| Kyperounta1B |  | 55764 | 50140 | 3.766 | 0.881 | 151 | 131 |
| Kyperounta2A |  | 61686 | 55307 | 3.731 | 0.867 | 149 | 129 |
| Kathikas1A | Post-fermentation | 41467 | 37234 | 3.742 | 0.864 | 124 | 112 |
| Kathikas1B |  | 59555 | 53086 | 3.414 | 0.829 | 154 | 123 |
| Kathikas2A |  | 38711 | 34974 | 3.392 | 0.838 | 128 | 114 |
| Kathikas2B |  | 19332 | 17411 | 3.330 | 0.843 | 83 | 82 |
| Koilani1A |  | 25531 | 22977 | 2.925 | 0.781 | 83 | 82 |
| Koilani2A |  | 24000 | 21784 | 3.256 | 0.817 | 95 | 93 |
| Koilani2B |  | 30766 | 27801 | 3.519 | 0.831 | 117 | 107 |
| Panayia 1A |  | 35604 | 31833 | 3.438 | 0.847 | 118 | 109 |
| Panayia 1B |  | 18763 | 16651 | 3.130 | 0.802 | 66 | 65 |
| Panayia 2A |  | 13475 | 12251 | 3.322 | 0.832 | 70 | 69 |
| Statos1A |  | 41092 | 37215 | 3.357 | 0.819 | 122 | 111 |
| Statos1B |  | 41361 | 37144 | 2.880 | 0.759 | 111 | 103 |
| Statos2A |  | 27945 | 25216 | 2.930 | 0.774 | 89 | 88 |
| Statos2B |  | 45645 | 41523 | 3.106 | 0.791 | 135 | 118 |
| Kyperounta1A |  | 56483 | 51236 | 3.604 | 0.844 | 130 | 114 |
| Kyperounta1B |  | 45758 | 41678 | 3.685 | 0.865 | 117 | 109 |
| Kyperounta2A |  | 34203 | 30014 | 3.029 | 0.765 | 84 | 81 |
| Kyperounta2B |  | 26859 | 23686 | 3.129 | 0.791 | 80 | 80 |

**Table S5**. Sample information, fungal alpha diversity indexes and observed OTUs for the variety Maratheftiko

| Sample-ID | Stage of fermentation | Reads passing filter | Denoised reads | Shannon | Simpson | Chao1 | Observed OTUs |
| --- | --- | --- | --- | --- | --- | --- | --- |
| Kathikas1A | Pre-fermentation | 18044 | 14666 | 4.278846 | 0.921398 | 71 | 67 |
| Kathikas1B |  | 12836 | 10631 | 4.331088 | 0.926529 | 56 | 55 |
| Kathikas2A |  | 24861 | 16200 | 3.807707 | 0.855454 | 101 | 92 |
| Kathikas2B |  | 8586 | 5520 | 3.432802 | 0.844072 | 52 | 52 |
| Koilani1A |  | 17113 | 15398 | 2.865887 | 0.798808 | 54 | 46 |
| Koilani1B |  | 21533 | 19275 | 2.881508 | 0.81128 | 48 | 39 |
| Koilani2A |  | 23799 | 14962 | 3.463951 | 0.825931 | 77 | 70 |
| Koilani2B |  | 21219 | 12701 | 3.987809 | 0.859836 | 96 | 93 |
| Panayia 1A |  | 15327 | 12857 | 3.911683 | 0.888403 | 84 | 73 |
| Panayia 1B |  | 27262 | 23209 | 3.810216 | 0.880048 | 109 | 93 |
| Panayia 2A |  | 17342 | 13475 | 4.60073 | 0.918852 | 107 | 101 |
| Panayia 2B |  | 16723 | 12973 | 4.534639 | 0.913696 | 120 | 114 |
| Statos1A |  | 47446 | 40365 | 4.299854 | 0.912705 | 144 | 108 |
| Statos1B |  | 19123 | 16515 | 4.341653 | 0.913494 | 116 | 101 |
| Statos2A |  | 28217 | 20232 | 3.858698 | 0.884283 | 69 | 64 |
| Statos2B |  | 25749 | 17936 | 3.845113 | 0.883965 | 69 | 61 |
| Kyperounta1A |  | 13775 | 8831 | 4.108369 | 0.884093 | 95 | 93 |
| Kyperounta1B |  | 20203 | 14028 | 3.57511 | 0.854618 | 103 | 81 |
| Kyperounta2A |  | 25196 | 12482 | 4.5221 | 0.883741 | 155 | 146 |
| Kyperounta2B |  | 18871 | 10185 | 3.95828 | 0.853687 | 113 | 107 |
| Kathikas1A | During fermentation | 16829 | 13247 | 3.199285 | 0.828959 | 52 | 48 |
| Kathikas1B |  | 15902 | 12317 | 3.236372 | 0.830146 | 53 | 52 |
| Kathikas2A |  | 23322 | 15931 | 2.513189 | 0.741262 | 40 | 34 |
| Kathikas2B |  | 29754 | 21581 | 2.521466 | 0.737717 | 43 | 37 |
| Koilani1A |  | 20930 | 16679 | 3.521198 | 0.867314 | 61 | 54 |
| Koilani2A |  | 25206 | 16710 | 3.484239 | 0.859402 | 66 | 62 |
| Koilani2B |  | 29983 | 6288 | 3.132743 | 0.807666 | 43 | 43 |
| Panayia 1A |  | 27282 | 24353 | 2.898346 | 0.830884 | 43 | 36 |
| Panayia 1B |  | 13956 | 11911 | 2.998681 | 0.845692 | 35 | 33 |
| Panayia 2A |  | 26661 | 22328 | 2.656278 | 0.763802 | 53 | 45 |
| Panayia 2B |  | 15142 | 12766 | 2.487663 | 0.737656 | 43 | 37 |
| Statos1A |  | 66441 | 39179 | 3.468744 | 0.871501 | 72 | 56 |
| Statos1B |  | 21246 | 12253 | 3.413541 | 0.867498 | 49 | 48 |
| Statos2A |  | 29115 | 12864 | 2.755992 | 0.748343 | 49 | 49 |
| Statos2B |  | 35091 | 10602 | 2.617625 | 0.731926 | 43 | 42 |
| Kyperounta1A |  | 37120 | 18946 | 2.947205 | 0.8 | 57 | 50 |
| Kyperounta1B |  | 69080 | 42308 | 3.200537 | 0.803863 | 136 | 94 |
| Kyperounta2A |  | 55959 | 28529 | 3.22454 | 0.800652 | 130 | 105 |
| Kyperounta2B |  | 20206 | 10838 | 3.554615 | 0.826868 | 97 | 95 |
| Kathikas1B | Post-fermentation | 47821 | 11735 | 3.438087 | 0.78463 | 53 | 43 |
| Kathikas2A |  | 67562 | 30819 | 4.049948 | 0.904865 | 79 | 64 |
| Kathikas2B |  | 66561 | 40073 | 3.703926 | 0.86848 | 80 | 58 |
| Koilani1A |  | 104602 | 19942 | 3.026023 | 0.799778 | 28 | 26 |
| Koilani1B |  | 19365 | 13911 | 3.911753 | 0.904461 | 62 | 53 |
| Koilani2A |  | 58077 | 44078 | 3.173941 | 0.843261 | 46 | 34 |
| Koilani2B |  | 20430 | 17292 | 2.798048 | 0.687729 | 65 | 46 |
| Panayia 1A |  | 39745 | 34230 | 3.062283 | 0.795344 | 80 | 51 |
| Panayia 1B |  | 41479 | 33781 | 2.921356 | 0.809945 | 50 | 37 |
| Panayia 2A |  | 21934 | 18940 | 2.872016 | 0.764703 | 66 | 48 |
| Panayia 2B |  | 44474 | 36749 | 2.931035 | 0.782868 | 72 | 46 |
| Statos1A |  | 69719 | 40827 | 3.536888 | 0.857109 | 72 | 54 |
| Statos1B |  | 43374 | 25734 | 3.567795 | 0.857598 | 57 | 47 |
| Statos2A |  | 57217 | 28025 | 3.222393 | 0.815829 | 85 | 56 |
| Statos2B |  | 31671 | 15254 | 2.477713 | 0.724624 | 51 | 39 |
| Kyperounta1A |  | 35967 | 16668 | 2.537386 | 0.737719 | 54 | 36 |
| Kyperounta1B |  | 47898 | 22748 | 2.554261 | 0.742992 | 71 | 39 |
| Kyperounta2A |  | 47372 | 22388 | 2.578913 | 0.747081 | 62 | 38 |
| Kyperounta2B |  | 41279 | 17105 | 2.782253 | 0.774666 | 59 | 43 |

**Table S6**. Comparison of the different terroirs alpha diversity (Shannon index) for fungal and bacterial communities for the two varieties (Xynisteri and Maratheftiko) during the stages of fermentation (pre-, during and post-fermentation) based on the Kruskal-Wallis tests

| **Microorganism** | **Wine Grape Variety** | **Stage of fermentation** | **Group 1** | **Group 2** | **H** | **p-value** | **q-value** |
| --- | --- | --- | --- | --- | --- | --- | --- |
| Fungus | Xynisteri | Pre-fermentation | Kathikas (n=4) | Koilani (n=4) | 5.333333 | 0.020921 | 0.05 |
|  |  |  | Kathikas (n=4) | Kyperounta (n=4) | 0.333333 | 0.563703 | 0.63 |
|  |  |  | Kathikas (n=4) | Panayia (n=4) | 0.75 | 0.386476 | 0.48 |
|  |  |  | Kathikas (n=4) | Statos (n=4) | 2.083333 | 0.148915 | 0.25 |
|  |  |  | Koilani (n=4) | Kyperounta (n=4) | 5.333333 | 0.020921 | 0.05 |
|  |  |  | Koilani (n=4) | Panayia (n=4) | 5.333333 | 0.020921 | 0.05 |
|  |  |  | Koilani (n=4) | Statos (n=4) | 5.333333 | 0.020921 | 0.05 |
|  |  |  | Kyperounta (n=4) | Panayia (n=4) | 1.333333 | 0.248213 | 0.35 |
|  |  |  | Kyperounta (n=4) | Statos (n=4) | 3 | 0.083265 | 0.17 |
|  |  |  | Panayia (n=4) | Statos (n=4) | 0.083333 | 0.77283 | 0.77 |
| Fungus | Xynisteri | During fermentation | Kathikas (n=4) | Koilani (n=4) | 2.083333 | 0.148915 | 0.25 |
|  |  |  | Kathikas (n=4) | Kyperounta (n=4) | 5.333333 | 0.020921 | 0.10 |
|  |  |  | Kathikas (n=4) | Panayia (n=4) | 0 | 1 | 1.00 |
|  |  |  | Kathikas (n=4) | Statos (n=4) | 0.333333 | 0.563703 | 0.70 |
|  |  |  | Koilani (n=4) | Kyperounta (n=4) | 3 | 0.083265 | 0.25 |
|  |  |  | Koilani (n=4) | Panayia (n=4) | 2.083333 | 0.148915 | 0.25 |
|  |  |  | Koilani (n=4) | Statos (n=4) | 0.083333 | 0.77283 | 0.86 |
|  |  |  | Kyperounta (n=4) | Panayia (n=4) | 5.333333 | 0.020921 | 0.10 |
|  |  |  | Kyperounta (n=4) | Statos (n=4) | 2.083333 | 0.148915 | 0.25 |
|  |  |  | Panayia (n=4) | Statos (n=4) | 0.333333 | 0.563703 | 0.70 |
| Fungus | Xynisteri | Post-fermentation | Kathikas (n=4) | Koilani (n=4) | 0.333333 | 0.563703 | 0.81 |
|  |  |  | Kathikas (n=4) | Kyperounta (n=4) | 3 | 0.083265 | 0.21 |
|  |  |  | Kathikas (n=4) | Panayia (n=4) | 5.333333 | 0.020921 | 0.10 |
|  |  |  | Kathikas (n=4) | Statos (n=4) | 1.333333 | 0.248213 | 0.41 |
|  |  |  | Koilani (n=4) | Kyperounta (n=4) | 3 | 0.083265 | 0.21 |
|  |  |  | Koilani (n=4) | Panayia (n=4) | 5.333333 | 0.020921 | 0.10 |
|  |  |  | Koilani (n=4) | Statos (n=4) | 0 | 1 | 1.00 |
|  |  |  | Kyperounta (n=4) | Panayia (n=4) | 0 | 1 | 1.00 |
|  |  |  | Kyperounta (n=4) | Statos (n=4) | 1.333333 | 0.248213 | 0.41 |
|  |  |  | Panayia (n=4) | Statos (n=4) | 0 | 1 | 1.00 |
| Fungus | Maratheftiko | Post-fermentation | Kathikas (n=4) | Koilani (n=4) | 2.083333 | 0.148915 | 0.37 |
|  |  |  | Kathikas (n=4) | Kyperounta (n=4) | 0.083333 | 0.77283 | 0.86 |
|  |  |  | Kathikas (n=4) | Panayia (n=4) | 1.333333 | 0.248213 | 0.50 |
|  |  |  | Kathikas (n=4) | Statos (n=4) | 0.75 | 0.386476 | 0.64 |
|  |  |  | Koilani (n=4) | Kyperounta (n=4) | 3 | 0.083265 | 0.28 |
|  |  |  | Koilani (n=4) | Panayia (n=4) | 3 | 0.083265 | 0.28 |
|  |  |  | Koilani (n=4) | Statos (n=4) | 3 | 0.083265 | 0.28 |
|  |  |  | Kyperounta (n=4) | Panayia (n=4) | 0.333333 | 0.563703 | 0.70 |
|  |  |  | Kyperounta (n=4) | Statos (n=4) | 0 | 1 | 1.00 |
|  |  |  | Panayia (n=4) | Statos (n=4) | 0.333333 | 0.563703 | 0.70 |
| Fungus | Maratheftiko | During fermentation | Kathikas (n=4) | Koilani (n=3) | 2 | 0.157299 | 0.39 |
|  |  |  | Kathikas (n=4) | Kyperounta (n=4) | 1.333333 | 0.248213 | 0.41 |
|  |  |  | Kathikas (n=4) | Panayia (n=4) | 0.333333 | 0.563703 | 0.63 |
|  |  |  | Kathikas (n=4) | Statos (n=4) | 1.333333 | 0.248213 | 0.41 |
|  |  |  | Koilani (n=3) | Kyperounta (n=4) | 0.125 | 0.723674 | 0.72 |
|  |  |  | Koilani (n=3) | Panayia (n=4) | 4.5 | 0.033895 | 0.22 |
|  |  |  | Koilani (n=3) | Statos (n=4) | 2 | 0.157299 | 0.39 |
|  |  |  | Kyperounta (n=4) | Panayia (n=4) | 4.083333 | 0.043308 | 0.22 |
|  |  |  | Kyperounta (n=4) | Statos (n=4) | 0.333333 | 0.563703 | 0.63 |
|  |  |  | Panayia (n=4) | Statos (n=4) | 0.75 | 0.386476 | 0.55 |
| Fungus | Maratheftiko | Post-fermentation | Kathikas (n=3) | Koilani (n=4) | 3 | 0.083265 | 0.14 |
|  |  |  | Kathikas (n=3) | Kyperounta (n=4) | 5.333333 | 0.020921 | 0.05 |
|  |  |  | Kathikas (n=3) | Panayia (n=4) | 5.333333 | 0.020921 | 0.05 |
|  |  |  | Kathikas (n=3) | Statos (n=4) | 3 | 0.083265 | 0.14 |
|  |  |  | Koilani (n=4) | Kyperounta (n=4) | 5.333333 | 0.020921 | 0.05 |
|  |  |  | Koilani (n=4) | Panayia (n=4) | 0.75 | 0.386476 | 0.43 |
|  |  |  | Koilani (n=4) | Statos (n=4) | 0.083333 | 0.77283 | 0.77 |
|  |  |  | Kyperounta (n=4) | Panayia (n=4) | 5.333333 | 0.020921 | 0.05 |
|  |  |  | Kyperounta (n=4) | Statos (n=4) | 1.333333 | 0.248213 | 0.31 |
|  |  |  | Panayia (n=4) | Statos (n=4) | 1.333333 | 0.248213 | 0.31 |
| Bacteria | Xynisteri | Pre-fermentation | Kathikas (n=4) | Koilani (n=4) | 3 | 0.083265 | 0.17 |
|  |  |  | Kathikas (n=4) | Kyperounta (n=4) | 5.333333 | 0.020921 | 0.14 |
|  |  |  | Kathikas (n=4) | Panayia (n=1) | 2 | 0.157299 | 0.20 |
|  |  |  | Kathikas (n=4) | Statos (n=4) | 4.083333 | 0.043308 | 0.14 |
|  |  |  | Koilani (n=4) | Kyperounta (n=4) | 4.083333 | 0.043308 | 0.14 |
|  |  |  | Koilani (n=4) | Panayia (n=1) | 2 | 0.157299 | 0.20 |
|  |  |  | Koilani (n=4) | Statos (n=4) | 0.333333 | 0.563703 | 0.63 |
|  |  |  | Kyperounta (n=4) | Panayia (n=1) | 0 | 1 | 1.00 |
|  |  |  | Kyperounta (n=4) | Statos (n=4) | 3 | 0.083265 | 0.17 |
|  |  |  | Panayia (n=1) | Statos (n=4) | 2 | 0.157299 | 0.20 |
| Bacteria | Xynisteri | During fermentation | Kathikas (n=3) | Koilani (n=4) | 0.125 | 0.723674 | 0.77 |
|  |  |  | Kathikas (n=3) | Kyperounta (n=4) | 0.125 | 0.723674 | 0.77 |
|  |  |  | Kathikas (n=3) | Panayia (n=4) | 1.125 | 0.288844 | 0.58 |
|  |  |  | Kathikas (n=3) | Statos (n=4) | 1.125 | 0.288844 | 0.58 |
|  |  |  | Koilani (n=4) | Kyperounta (n=4) | 1.333333 | 0.248213 | 0.58 |
|  |  |  | Koilani (n=4) | Panayia (n=4) | 0.083333 | 0.77283 | 0.77 |
|  |  |  | Koilani (n=4) | Statos (n=4) | 0.333333 | 0.563703 | 0.77 |
|  |  |  | Kyperounta (n=4) | Panayia (n=4) | 3 | 0.083265 | 0.42 |
|  |  |  | Kyperounta (n=4) | Statos (n=4) | 5.333333 | 0.020921 | 0.21 |
|  |  |  | Panayia (n=4) | Statos (n=4) | 0.083333 | 0.77283 | 0.77 |
| Bacteria | Xynisteri | Post-fermentation | Kathikas (n=4) | Koilani (n=4) | 0.083333 | 0.77283 | 0.97 |
|  |  |  | Kathikas (n=4) | Kyperounta (n=4) | 0.083333 | 0.77283 | 0.97 |
|  |  |  | Kathikas (n=4) | Panayia (n=4) | 0.75 | 0.386476 | 0.96 |
|  |  |  | Kathikas (n=4) | Statos (n=3) | 0.125 | 0.723674 | 0.97 |
|  |  |  | Koilani (n=4) | Kyperounta (n=4) | 0 | 1 | 1.00 |
|  |  |  | Koilani (n=4) | Panayia (n=4) | 3 | 0.083265 | 0.42 |
|  |  |  | Koilani (n=4) | Statos (n=3) | 0.5 | 0.4795 | 0.96 |
|  |  |  | Kyperounta (n=4) | Panayia (n=4) | 3 | 0.083265 | 0.42 |
|  |  |  | Kyperounta (n=4) | Statos (n=3) | 0 | 1 | 1.00 |
|  |  |  | Panayia (n=4) | Statos (n=3) | 2 | 0.157299 | 0.52 |
| Bacteria | Maratheftiko | Pre-fermentation | Kathikas (n=3) | Koilani (n=4) | 3.125 | 0.0771 | 0.26 |
|  |  |  | Kathikas (n=3) | Kyperounta (n=4) | 0.125 | 0.723674 | 0.72 |
|  |  |  | Kathikas (n=3) | Panayia (n=3) | 0.428571 | 0.512691 | 0.57 |
|  |  |  | Kathikas (n=3) | Statos (n=4) | 0.5 | 0.4795 | 0.57 |
|  |  |  | Koilani (n=4) | Kyperounta (n=4) | 2.083333 | 0.148915 | 0.31 |
|  |  |  | Koilani (n=4) | Panayia (n=3) | 3.125 | 0.0771 | 0.26 |
|  |  |  | Koilani (n=4) | Statos (n=4) | 5.333333 | 0.020921 | 0.21 |
|  |  |  | Kyperounta (n=4) | Panayia (n=3) | 1.125 | 0.288844 | 0.48 |
|  |  |  | Kyperounta (n=4) | Statos (n=4) | 0.75 | 0.386476 | 0.55 |
|  |  |  | Panayia (n=3) | Statos (n=4) | 2 | 0.157299 | 0.31 |
| Bacteria | Maratheftiko | During fermentation | Kathikas (n=3) | Koilani (n=2) | 0 | 1 | 1.00 |
|  |  |  | Kathikas (n=3) | Kyperounta (n=3) | 2.333333 | 0.12663 | 0.25 |
|  |  |  | Kathikas (n=3) | Panayia (n=3) | 0.047619 | 0.827259 | 0.92 |
|  |  |  | Kathikas (n=3) | Statos (n=4) | 0.5 | 0.4795 | 0.69 |
|  |  |  | Koilani (n=2) | Kyperounta (n=3) | 3 | 0.083265 | 0.21 |
|  |  |  | Koilani (n=2) | Panayia (n=3) | 0.333333 | 0.563703 | 0.70 |
|  |  |  | Koilani (n=2) | Statos (n=4) | 1.928571 | 0.164915 | 0.27 |
|  |  |  | Kyperounta (n=3) | Panayia (n=3) | 3.857143 | 0.049535 | 0.21 |
|  |  |  | Kyperounta (n=3) | Statos (n=4) | 3.125 | 0.0771 | 0.21 |
|  |  |  | Panayia (n=3) | Statos (n=4) | 3.125 | 0.0771 | 0.21 |
| Bacteria | Maratheftiko | Post-fermentation | Kathikas (n=4) | Koilani (n=3) | 1.125 | 0.288844 | 0.72 |
|  |  |  | Kathikas (n=4) | Kyperounta (n=4) | 0.333333 | 0.563703 | 0.80 |
|  |  |  | Kathikas (n=4) | Panayia (n=2) | 0.214286 | 0.643429 | 0.80 |
|  |  |  | Kathikas (n=4) | Statos (n=4) | 4.083333 | 0.043308 | 0.43 |
|  |  |  | Koilani (n=3) | Kyperounta (n=4) | 0.5 | 0.4795 | 0.80 |
|  |  |  | Koilani (n=3) | Panayia (n=2) | 0 | 1 | 1.00 |
|  |  |  | Koilani (n=3) | Statos (n=4) | 0.5 | 0.4795 | 0.80 |
|  |  |  | Kyperounta (n=4) | Panayia (n=2) | 0 | 1 | 1.00 |
|  |  |  | Kyperounta (n=4) | Statos (n=4) | 2.083333 | 0.148915 | 0.55 |
|  |  |  | Panayia (n=2) | Statos (n=4) | 1.928571 | 0.164915 | 0.55 |

**Table S7**. Comparison of the different terroirs’ beta diversity (Bray-Curtis dissimilarity) for fungal and bacterial communities for the two varieties (Xynisteri and Maratheftiko) during the stages of fermentation (pre-, during and post-fermentation) based on the permanova-pairwise test.

| Microorganism | Variety | Stage of fermentation | Group 1 | Group 2 | Sample size | Permutations | pseudo-F | p-value | q-value |
| --- | --- | --- | --- | --- | --- | --- | --- | --- | --- |
| Fungus | Xynisteri | Pre-fermentation | Kathikas | Koilani | 7 | 999 | 1.782758 | 0.035 | 0.04 |
|  |  |  | Kathikas | Kyperounta | 7 | 999 | 4.031437 | 0.017 | 0.04 |
|  |  |  | Kathikas | Panayia | 6 | 999 | 2.07873 | 0.028 | 0.04 |
|  |  |  | Kathikas | Statos | 5 | 999 | 2.806316 | 0.016 | 0.04 |
|  |  |  | Koilani | Kyperounta | 8 | 999 | 4.268398 | 0.027 | 0.04 |
|  |  |  | Koilani | Panayia | 7 | 999 | 1.861344 | 0.03 | 0.04 |
|  |  |  | Koilani | Statos | 6 | 999 | 2.258973 | 0.029 | 0.04 |
|  |  |  | Kyperounta | Panayia | 7 | 999 | 3.219195 | 0.029 | 0.04 |
|  |  |  | Kyperounta | Statos | 6 | 999 | 3.992202 | 0.031 | 0.04 |
|  |  |  | Panayia | Statos | 5 | 999 | 1.030794 | 0.302 | 0.30 |
|  |  | During Fermentation | Kathikas | Koilani | 8 | 999 | 1.300239 | 0.261 | 0.26 |
|  |  |  | Kathikas | Kyperounta | 8 | 999 | 3.368595 | 0.023 | 0.05 |
|  |  |  | Kathikas | Panayia | 8 | 999 | 2.051224 | 0.028 | 0.05 |
|  |  |  | Kathikas | Statos | 8 | 999 | 2.419856 | 0.033 | 0.05 |
|  |  |  | Koilani | Kyperounta | 8 | 999 | 4.477297 | 0.043 | 0.05 |
|  |  |  | Koilani | Panayia | 8 | 999 | 3.908091 | 0.027 | 0.05 |
|  |  |  | Koilani | Statos | 8 | 999 | 3.614666 | 0.039 | 0.05 |
|  |  |  | Kyperounta | Panayia | 8 | 999 | 5.496033 | 0.018 | 0.05 |
|  |  |  | Kyperounta | Statos | 8 | 999 | 4.510562 | 0.029 | 0.05 |
|  |  |  | Panayia | Statos | 8 | 999 | 3.833735 | 0.03 | 0.05 |
|  |  | Post-fermentation | Kathikas | Koilani | 8 | 999 | 2.907016 | 0.029 | 0.04 |
|  |  |  | Kathikas | Kyperounta | 8 | 999 | 3.265458 | 0.031 | 0.04 |
|  |  |  | Kathikas | Panayia | 8 | 999 | 1.286972 | 0.074 | 0.07 |
|  |  |  | Kathikas | Statos | 8 | 999 | 2.118906 | 0.029 | 0.04 |
|  |  |  | Koilani | Kyperounta | 8 | 999 | 3.657774 | 0.025 | 0.04 |
|  |  |  | Koilani | Panayia | 8 | 999 | 3.302765 | 0.03 | 0.04 |
|  |  |  | Koilani | Statos | 8 | 999 | 2.360598 | 0.029 | 0.04 |
|  |  |  | Kyperounta | Panayia | 8 | 999 | 3.037067 | 0.036 | 0.04 |
|  |  |  | Kyperounta | Statos | 8 | 999 | 2.409568 | 0.018 | 0.04 |
|  |  |  | Panayia | Statos | 8 | 999 | 1.924059 | 0.022 | 0.04 |
|  | Maratheftiko | Pre-fermentation | Kathikas | Koilani | 8 | 999 | 0.703314 | 0.672 | 0.67 |
|  |  |  | Kathikas | Kyperounta | 8 | 999 | 2.696088 | 0.024 | 0.07 |
|  |  |  | Kathikas | Panayia | 8 | 999 | 1.883401 | 0.062 | 0.08 |
|  |  |  | Kathikas | Statos | 8 | 999 | 2.247709 | 0.024 | 0.07 |
|  |  |  | Koilani | Kyperounta | 8 | 999 | 2.536336 | 0.027 | 0.07 |
|  |  |  | Koilani | Panayia | 8 | 999 | 1.918871 | 0.051 | 0.08 |
|  |  |  | Koilani | Statos | 8 | 999 | 1.98687 | 0.05 | 0.08 |
|  |  |  | Kyperounta | Panayia | 8 | 999 | 2.408806 | 0.039 | 0.07 |
|  |  |  | Kyperounta | Statos | 8 | 999 | 3.821177 | 0.034 | 0.07 |
|  |  |  | Panayia | Statos | 8 | 999 | 1.847104 | 0.055 | 0.08 |
|  |  | During Fermentation | Kathikas | Koilani | 7 | 999 | 2.059919 | 0.054 | 0.08 |
|  |  |  | Kathikas | Kyperounta | 8 | 999 | 2.784993 | 0.025 | 0.05 |
|  |  |  | Kathikas | Panayia | 8 | 999 | 1.508567 | 0.116 | 0.13 |
|  |  |  | Kathikas | Statos | 8 | 999 | 2.37419 | 0.049 | 0.08 |
|  |  |  | Koilani | Kyperounta | 7 | 999 | 2.761519 | 0.026 | 0.05 |
|  |  |  | Koilani | Panayia | 7 | 999 | 1.647077 | 0.09 | 0.11 |
|  |  |  | Koilani | Statos | 7 | 999 | 1.660201 | 0.127 | 0.13 |
|  |  |  | Kyperounta | Panayia | 8 | 999 | 2.760677 | 0.026 | 0.05 |
|  |  |  | Kyperounta | Statos | 8 | 999 | 4.229872 | 0.026 | 0.05 |
|  |  |  | Panayia | Statos | 8 | 999 | 2.989717 | 0.024 | 0.05 |
|  |  | Post-fermentation | Kathikas | Koilani | 7 | 999 | 1.271314 | 0.215 | 0.27 |
|  |  |  | Kathikas | Kyperounta | 7 | 999 | 2.238834 | 0.032 | 0.08 |
|  |  |  | Kathikas | Panayia | 7 | 999 | 2.193815 | 0.019 | 0.08 |
|  |  |  | Kathikas | Statos | 7 | 999 | 1.033199 | 0.401 | 0.44 |
|  |  |  | Koilani | Kyperounta | 8 | 999 | 3.154768 | 0.031 | 0.08 |
|  |  |  | Koilani | Panayia | 8 | 999 | 1.601111 | 0.114 | 0.16 |
|  |  |  | Koilani | Statos | 8 | 999 | 1.00355 | 0.442 | 0.44 |
|  |  |  | Kyperounta | Panayia | 8 | 999 | 3.05104 | 0.027 | 0.08 |
|  |  |  | Kyperounta | Statos | 8 | 999 | 2.214931 | 0.041 | 0.08 |
|  |  |  | Panayia | Statos | 8 | 999 | 1.394234 | 0.115 | 0.16 |
| Bacteria | Ksinisteri | Pre-fermentation | Kathikas | Koilani | 8 | 999 | 1.321954 | 0.087 | 0.22 |
|  |  |  | Kathikas | Kyperounta | 8 | 999 | 2.270437 | 0.026 | 0.09 |
|  |  |  | Kathikas | Panayia | 5 | 999 | 1.066594 | 0.599 | 0.60 |
|  |  |  | Kathikas | Statos | 8 | 999 | 1.035744 | 0.464 | 0.58 |
|  |  |  | Koilani | Kyperounta | 8 | 999 | 2.331258 | 0.027 | 0.09 |
|  |  |  | Koilani | Panayia | 5 | 999 | 0.946399 | 0.604 | 0.60 |
|  |  |  | Koilani | Statos | 8 | 999 | 1.300018 | 0.163 | 0.33 |
|  |  |  | Kyperounta | Panayia | 5 | 999 | 1.76532 | 0.223 | 0.33 |
|  |  |  | Kyperounta | Statos | 8 | 999 | 2.366102 | 0.028 | 0.09 |
|  |  |  | Panayia | Statos | 5 | 999 | 1.384136 | 0.228 | 0.33 |
|  |  | During Fermentation | Kathikas | Koilani | 7 | 999 | 1.05961 | 0.271 | 0.39 |
|  |  |  | Kathikas | Kyperounta | 7 | 999 | 1.097121 | 0.31 | 0.39 |
|  |  |  | Kathikas | Panayia | 7 | 999 | 1.008818 | 0.485 | 0.49 |
|  |  |  | Kathikas | Statos | 7 | 999 | 1.051586 | 0.4 | 0.44 |
|  |  |  | Koilani | Kyperounta | 8 | 999 | 1.539439 | 0.067 | 0.36 |
|  |  |  | Koilani | Panayia | 8 | 999 | 1.236665 | 0.234 | 0.39 |
|  |  |  | Koilani | Statos | 8 | 999 | 1.190502 | 0.147 | 0.36 |
|  |  |  | Kyperounta | Panayia | 8 | 999 | 1.386179 | 0.109 | 0.36 |
|  |  |  | Kyperounta | Statos | 8 | 999 | 1.3723 | 0.157 | 0.36 |
|  |  |  | Panayia | Statos | 8 | 999 | 1.249945 | 0.178 | 0.36 |
|  |  | Post-fermentation | Kathikas | Koilani | 8 | 999 | 0.9467 | 0.534 | 0.74 |
|  |  |  | Kathikas | Kyperounta | 8 | 999 | 1.088407 | 0.342 | 0.44 |
|  |  |  | Kathikas | Panayia | 8 | 999 | 1.085963 | 0.439 | 0.44 |
|  |  |  | Kathikas | Statos | 7 | 999 | 0.871909 | 0.699 | 0.74 |
|  |  |  | Koilani | Kyperounta | 8 | 999 | 1.160017 | 0.281 | 0.44 |
|  |  |  | Koilani | Panayia | 8 | 999 | 1.149728 | 0.355 | 0.44 |
|  |  |  | Koilani | Statos | 7 | 999 | 1.011781 | 0.48 | 0.83 |
|  |  |  | Kyperounta | Panayia | 8 | 999 | 1.490859 | 0.154 | 0.44 |
|  |  |  | Kyperounta | Statos | 7 | 999 | 1.444798 | 0.107 | 0.44 |
|  |  |  | Panayia | Statos | 7 | 999 | 1.274169 | 0.141 | 0.44 |
|  | Maratheftiko | Pre-fermentation | Kathikas | Koilani | 5 | 999 | 0.878244 | 1 | 0.67 |
|  |  |  | Kathikas | Kyperounta | 6 | 999 | 1.356204 | 0.102 | 0.52 |
|  |  |  | Kathikas | Panayia | 6 | 999 | 1.100768 | 0.321 | 0.63 |
|  |  |  | Kathikas | Statos | 7 | 999 | 1.69265 | 0.056 | 0.52 |
|  |  |  | Koilani | Kyperounta | 5 | 999 | 1.41705 | 0.099 | 0.52 |
|  |  |  | Koilani | Panayia | 5 | 999 | 1.229268 | 0.297 | 0.68 |
|  |  |  | Koilani | Statos | 6 | 999 | 1.828511 | 0.076 | 0.63 |
|  |  |  | Kyperounta | Panayia | 6 | 999 | 1.58288 | 0.217 | 0.52 |
|  |  |  | Kyperounta | Statos | 7 | 999 | 1.299252 | 0.183 | 0.52 |
|  |  |  | Panayia | Statos | 7 | 999 | 1.976459 | 0.204 | 0.52 |
|  |  | During Fermentation | Kathikas | Koilani | 7 | 999 | 0.955664 | 0.598 | 0.19 |
|  |  |  | Kathikas | Kyperounta | 8 | 999 | 1.584185 | 0.053 | 0.19 |
|  |  |  | Kathikas | Panayia | 6 | 999 | 0.876386 | 0.843 | 0.19 |
|  |  |  | Kathikas | Statos | 8 | 999 | 1.107314 | 0.346 | 1.00 |
|  |  |  | Koilani | Kyperounta | 7 | 999 | 1.65227 | 0.066 | 1.00 |
|  |  |  | Koilani | Panayia | 5 | 999 | 0.923636 | 0.506 | 1.00 |
|  |  |  | Koilani | Statos | 7 | 999 | 1.458292 | 0.079 | 0.19 |
|  |  |  | Kyperounta | Panayia | 6 | 999 | 1.116506 | 0.397 | 1.00 |
|  |  |  | Kyperounta | Statos | 8 | 999 | 1.583377 | 0.094 | 0.19 |
|  |  |  | Panayia | Statos | 6 | 999 | 1.111865 | 0.388 | 0.20 |
|  |  | Post-fermentation | Kathikas | Koilani | 7 | 999 | 0.955664 | 0.598 | 0.66 |
|  |  |  | Kathikas | Kyperounta | 8 | 999 | 1.584185 | 0.053 | 0.24 |
|  |  |  | Kathikas | Panayia | 6 | 999 | 0.876386 | 0.843 | 0.84 |
|  |  |  | Kathikas | Statos | 8 | 999 | 1.107314 | 0.346 | 0.57 |
|  |  |  | Koilani | Kyperounta | 7 | 999 | 1.65227 | 0.066 | 0.24 |
|  |  |  | Koilani | Panayia | 5 | 999 | 0.923636 | 0.506 | 0.63 |
|  |  |  | Koilani | Statos | 7 | 999 | 1.458292 | 0.079 | 0.24 |
|  |  |  | Kyperounta | Panayia | 6 | 999 | 1.116506 | 0.397 | 0.57 |
|  |  |  | Kyperounta | Statos | 8 | 999 | 1.583377 | 0.094 | 0.24 |
|  |  |  | Panayia | Statos | 6 | 999 | 1.111865 | 0.388 | 0.57 |

**Table S8**. Comparison of the two grape wine varieties Xynisteri and Maratheftiko microbial beta diversity (Bray-Curtis dissimilarity) during the stages of fermentation (pre-, during and post-fermentation) based on the permanova-pairwise test.

| Microorganism | Stage of fermentation | Variety 1 | Variety 2 | Sample size | Permutations | pseudo-F | p-value | q-value |
| --- | --- | --- | --- | --- | --- | --- | --- | --- |
| Fungus | Pre-fermentation | Maratheftiko | Xynisteri | 40 | 999 | 1.6226747 | 0.032 | 0.03 |
|  | During fermentation |  |  | 39 | 999 | 1.4905397 | 0.078 | 0.08 |
|  | Post-fermentation |  |  | 39 | 999 | 2.8008757 | 0.001 | 0.001 |
| Bacteria | Pre-fermentation |  |  | 35 | 999 | 1.459813 | 0.037 | 0.04 |
|  | During fermentation |  |  | 34 | 999 | 1.2400993 | 0.094 | 0.09 |
|  | Post-fermentation |  |  | 37 | 999 | 2.1189834 | 0.002 | 0.002 |

**Table S9**. Values that samples were rarefied, according to each comparison performed.

| Microorganism | Stage of fermentation | Variety | Rarefaction value |
| --- | --- | --- | --- |
| Bacteria | Pre-fermentation | Xynisteri | 15651 |
|  | During fermentation |  | 11206 |
|  | Post-fermentation |  | 18003 |
| Bacteria | Pre-fermentation | Maratheftiko | 12473 |
|  | During fermentation |  | 13757 |
|  | Post-fermentation |  | 12251 |
| Fungus | Pre-fermentation | Xynisteri | 5781 |
|  | During fermentation |  | 5472 |
|  | Post-fermentation |  | 9516 |
| Fungus | Pre-fermentation | Maratheftiko | 5520 |
|  | During fermentation |  | 6288 |
|  | Post-fermentation |  | 11735 |
| Bacteria | Pre-fermentation | Xynisteri/Maratheftiko | 12473 |
|  | During fermentation |  | 11206 |
|  | Post-fermentation |  | 12251 |
| Fungus | Pre-fermentation |  | 5520 |
|  | During fermentation |  | 5472 |
|  | Post-fermentation |  | 6288 |
